# Understanding complex genetic architecture of rice grain weight through QTL-meta analysis and candidate gene identification

**DOI:** 10.1038/s41598-022-17402-w

**Published:** 2022-08-16

**Authors:** C. Anilkumar, Rameswar Prasad Sah, T. P. Muhammed Azharudheen, Sasmita Behera, Namita Singh, Nitish Ranjan Prakash, N. C. Sunitha, B. N. Devanna, B. C. Marndi, B. C. Patra, Sunil Kumar Nair

**Affiliations:** 1 ICAR-National Rice Research Institute, Cuttack, India; 2 Indira Gandhi Krishi Vishwavidyalaya, Raipur, India; 3 ICAR-Central Soil Salinity Research Institute, Regional Research Station, Canning Town, India; 4 University of Agricultural Sciences, Bangalore, India

**Keywords:** Plant biotechnology, Plant breeding, Plant development, Plant genetics, Plant molecular biology

## Abstract

Quantitative trait loci (QTL) for rice grain weight identified using bi-parental populations in various environments were found inconsistent and have a modest role in marker assisted breeding and map-based cloning programs. Thus, the identification of a consistent consensus QTL region across populations is critical to deploy in marker aided breeding programs. Using the QTL meta-analysis technique, we collated rice grain weight QTL information from numerous studies done across populations and in diverse environments to find constitutive QTL for grain weight. Using information from 114 original QTL in meta-analysis, we discovered three significant Meta-QTL (MQTL) for grain weight on chromosome 3. According to gene ontology, these three MQTL have 179 genes, 25 of which have roles in developmental functions. Amino acid sequence BLAST of these genes indicated their orthologue conservation among core cereals with similar functions. MQTL3.1 includes the *OsAPX1*, *PDIL*, *SAUR*, and *OsASN1* genes, which are involved in grain development and have been discovered to play a key role in asparagine biosynthesis and metabolism, which is crucial for source-sink regulation. Five potential candidate genes were identified and their expression analysis indicated a significant role in early grain development. The gene sequence information retrieved from the 3 K rice genome project revealed the deletion of six bases coding for serine and alanine in the last exon of *OsASN1* led to an interruption in the synthesis of α-helix of the protein, which negatively affected the asparagine biosynthesis pathway in the low grain weight genotypes. Further, the MQTL3.1 was validated using linked marker RM7197 on a set of genotypes with extreme phenotypes. MQTL that have been identified and validated in our study have significant scope in MAS breeding and map-based cloning programs for improving rice grain weight.

## Introduction

Grain weight in rice is conventionally measured with one ‘thousand grains weight (TGW)’ and considered as a critical grain yield component trait. The TGW is influenced by associated grain traits like grain length, grain width, and thickness^1^. These traits are genetically controlled by many minor/major genes and are quantitatively inherited through generations. Over decades, researchers focused their investigations on grain weight to improve the grain yield in rice^2,3^. With the advent of molecular breeding technologies, plant breeding and botanical views have gained a deeper grasp of genetic control of TGW^4,5^. Application of molecular markers and linkage mapping techniques paved the path for the identification of genetic loci controlling TGW. In the last two decades, more than a hundred major effect quantitative trait loci (QTL) exclusively controlling TGW have been mapped using different bi-parental populations over various environmental conditions. However, only a few of them have been validated and cloned using isogenic lines^6^. Generally, QTL detected in different populations evaluated under different environments exhibit differential effects of phenotypic variance that create ambiguity in exploiting them in marker-assisted breeding programs. Further, bi-parental population based QTL identification is strongly influenced by contrasting parents, type of mapping population, size of population, growing environment, and choice of marker system that hinders application of these QTLs in breeding programs^7,8^.

QTL identified through bi-parental populations play a very modest role in rice breeding due to inconsistencies in growing environments, leading to discontinuities in their use^9^. Thus, identification of constitutive, robust and large-effect QTL over environments and across populations is critical for deployment in genomics assisted breeding programs^10^. Impact of G × E interaction, may bring change in level of correlation between phenotype and underlying QTL across environments^11^. This is also augmented with effects from differed genetic backgrounds^12,13^, thereby limiting their utilization. The efficacy of identified QTL in genomics-assisted breeding is further hampered by undesirable epistatic and modifier effects of distinct genetic backgrounds^14^. The possible reasons behind the inability to use such identified QTL are their implausible genetic predictions (with low LOD scores, predicted in small populations, faulty genotyping, limited environmental evaluation, etc.) and ignorance of interaction effects of QTLs, which are important in determining QTL stability. Hence, it is important to identify a trait-related consensus genomic region that includes more than one QTL and is surrogated by a single marker^15^. Such genomic regions are more useful to incorporate at least one or a few alleles from any of the constituent QTL to improve the target trait. Although, several QTL for grain weight characters in rice have been identified; leveraging the available information to enhance the insights of the underlying genetic mechanisms is more appropriate.

A computational technique, Meta-QTL (MQTL) analysis, which combines the reported QTL information precisely, has been developed and deployed to refine QTL positions by constructing a consensus map^16,17^. The consensus QTL predicted using meta-analysis of a group of QTL with a confidence interval of 95% is denoted as meta-QTL. These MQTL show high consistency, a small confidence interval, and a major effect on traits that can be effectively utilized in MAS breeding programs upon their validation in a set of germplasm accessions. To date, only a few MQTL studies have been reported in rice for grain yield^18,19^, plant characters^20,21^ and yield under drought stress^14^. Further, these studies were done by considering more than one trait (meta-trait), which is generally contributed by several component traits. Such analysis cannot conclusively describe the genetic and molecular mechanisms of component traits^22^. Hence, no meta-analysis of genetic factors associated with exclusive evidence on grain weight alone is available.

In this milieu, the present study was hypothesized the presence of consistent large effect QTL controlling grain weight on rice genome and tested the hypothesis by genome-wide meta-analysis with objectives, (1) prediction of MQTL exclusively for grain weight considering reported QTL, (2) identification of peak (tightly linked) molecular markers associated with MQTL, (3) validation of identified MQTL using linked peak markers in a set of genotypes comprising of two extreme phenotypes and (4) identification of candidate genes in the MQTL interval and predict their possible biological, molecular and cellular functions in rice grain weight development.

## Results

### Identification of MQTL for grain weight

**Figure 1:**
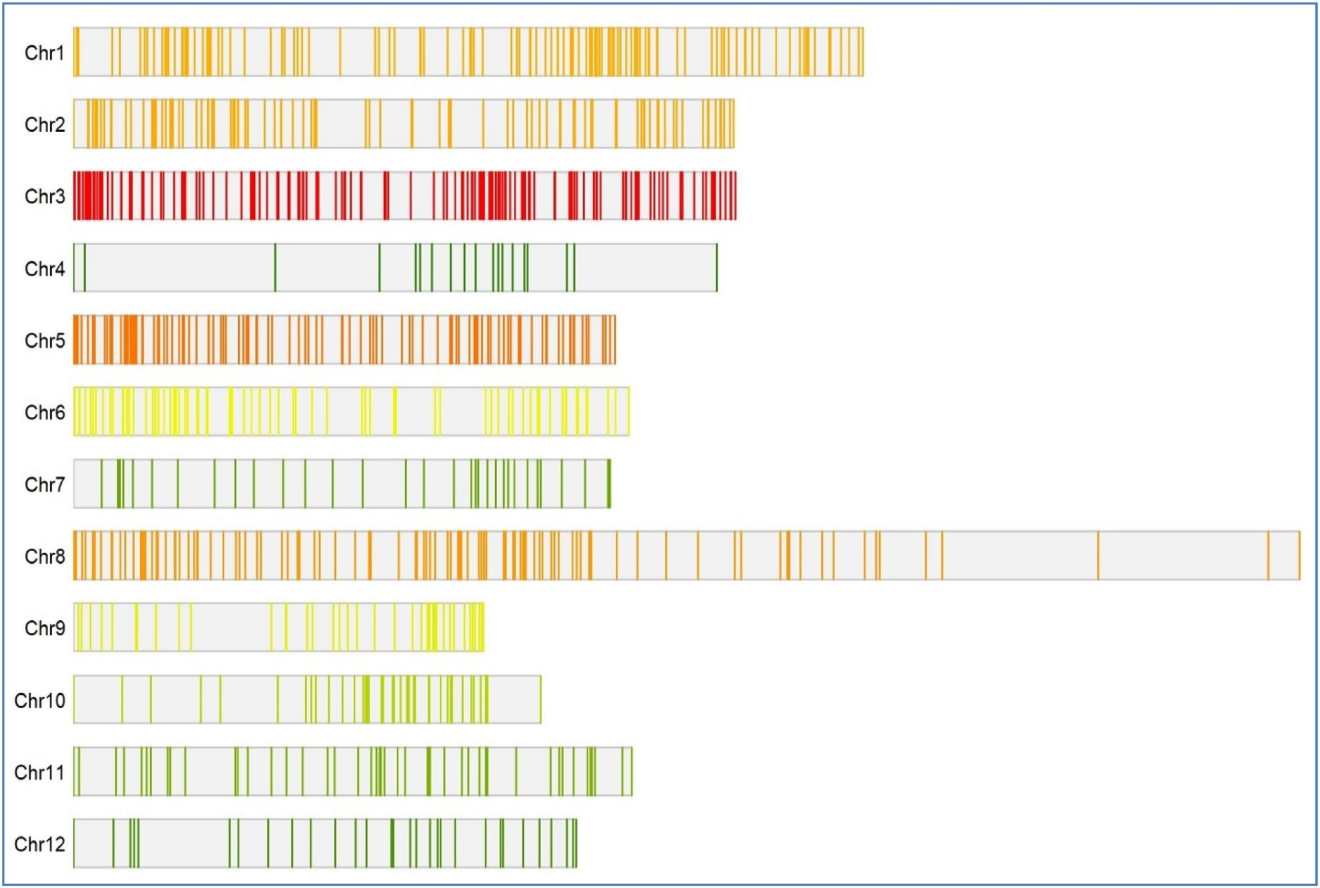
Distribution of the markers on the rice consensus map constructed and utilized for meta-QTL analysis in the present study (Color intensity from white to red indicate low to high density of marker, respectively).

**Figure 2:**
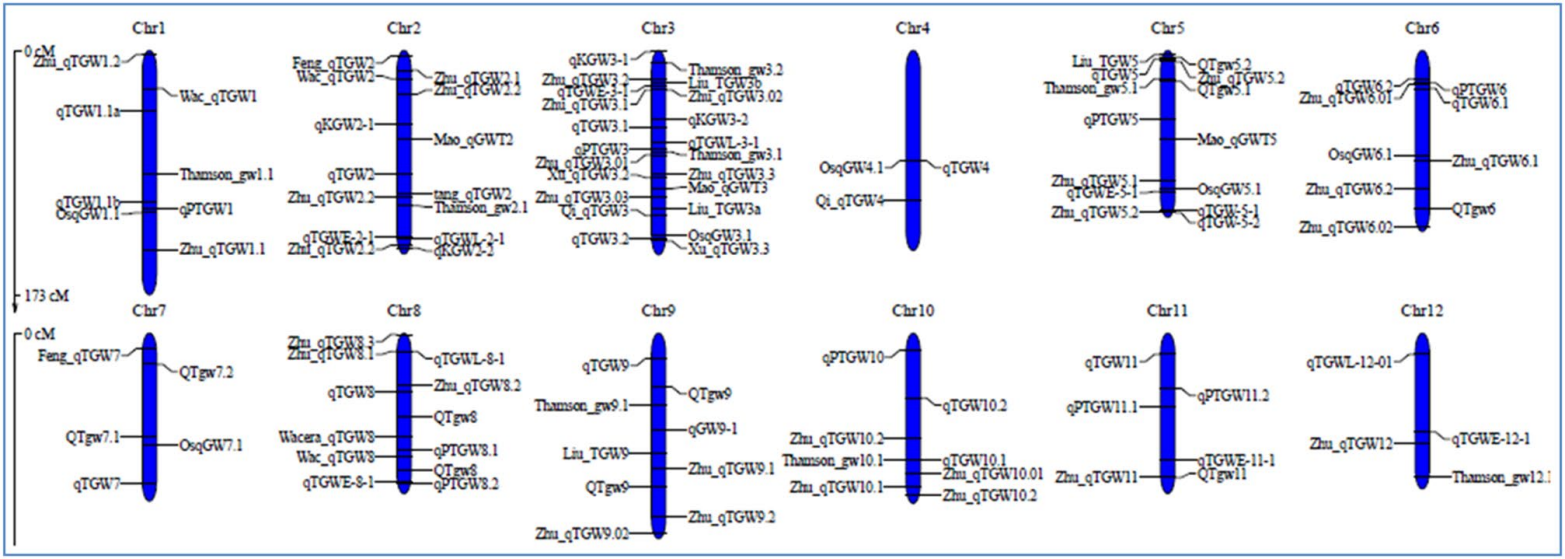
Distribution of reported grain weight QTL on all 12 rice chromosomes (name of the first author of original report is used as prefix while naming the QTL in this study).

**Figure 3:**
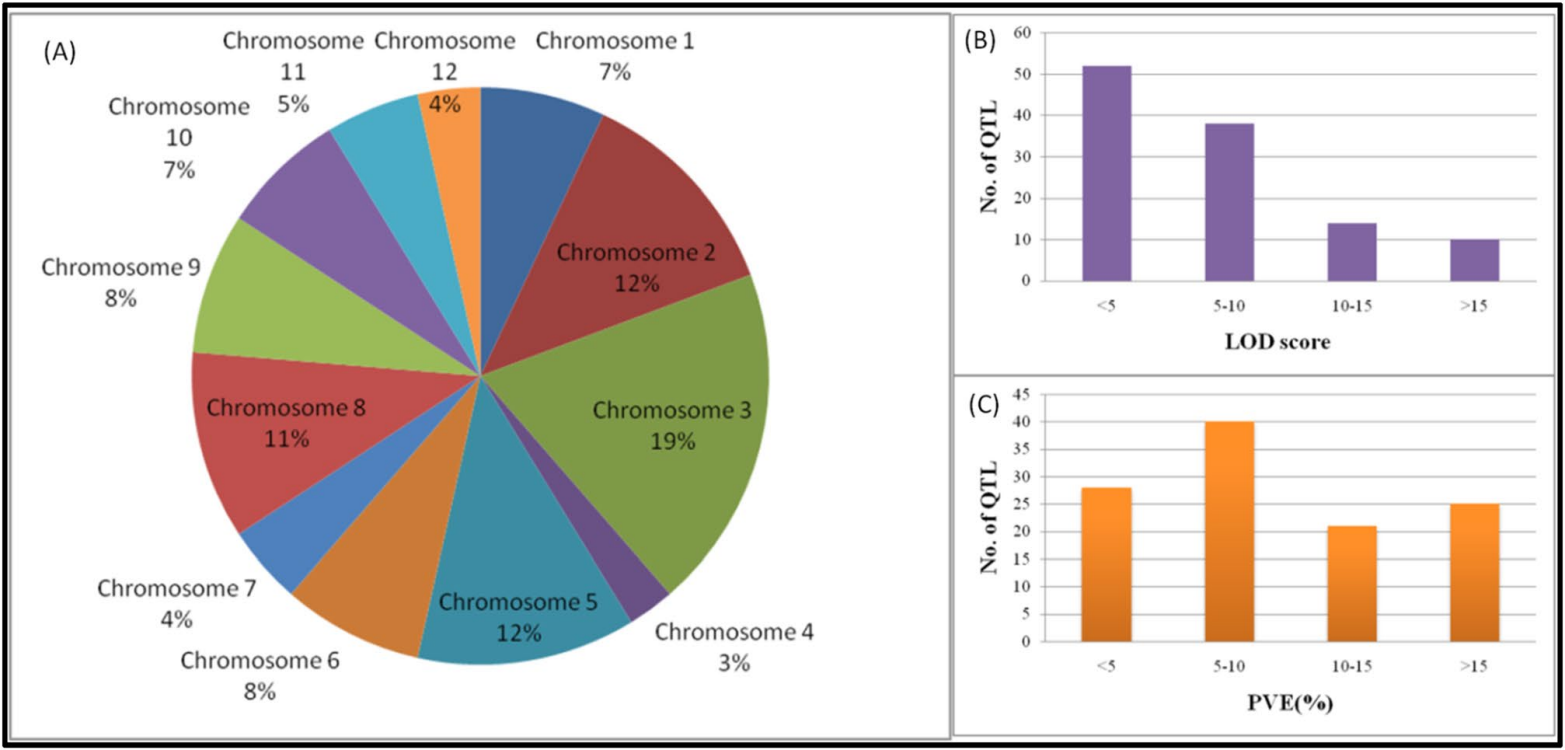
Detailed information on initial QTL utilized for QTL-meta analysis (**A**) chromosome distribution of initial QTL (**B**) LOD score information of initial QTL and (C) information on phynotypic variation explained by initial QTL considered for the study.

The information from 114 QTL found in 22 distinct independent research undertaken over the previous two decades was used to create the consensus genetic map (Supplementary file 1). The consensus map consisted of 1272 SSR markers evenly distributed over all 12 rice chromosomes (Fig. 1). While gathering the information, every precaution was made to ensure that only thousand grain weight specific QTL were extracted and no other seed shape related features were taken into account. Among all the QTL mapping studies included in the analysis, the mapping populations included a minimum of 39 and a maximum of 353 individuals tested under various environments. The number of QTL found in each investigation ranged from 3 to 22 and were spread across all 12 rice chromosomes (Fig. 2). The most QTL were found on chromosome 3 (22 QTL), whereas the fewest were found on chromosome 12 (4 QTL) (Fig. 3A). Further, the 114 QTL analyzed were grouped based on PVE (%) and LOD score. Almost 49% of the initial QTL were found to have a LOD score less than 5 (Fig. 3B). The PVE (%) of all 114 QTL ranges from 0.4 to 60% with a mean of 10.8%, with nearly 50% of them having a PVE (%) between 5 and 10% (Fig. 3C). After combining individual maps from 22 reported experiments, a consensus genetic map was created.

**Figure 4:**
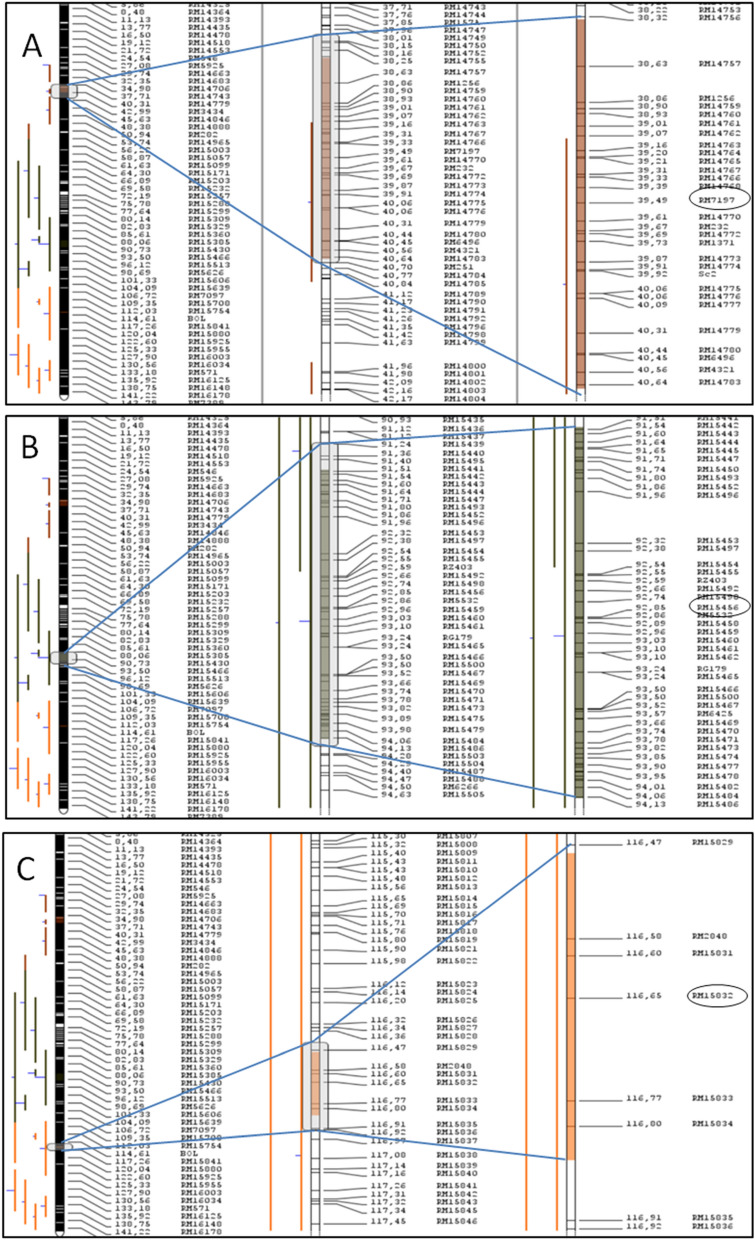
Magnified view of significant meta-QTL identified in the study (**A**) MQTL3.1 having interval of 0.49 Mb with peak marker RM7197, (**B**) MQTL3.2 having interval of 0.63 Mb with peak marker RM15456 and (**C**) MQTL3.3 having interval of 0.08 Mb with peak linked marker RM15832.

According to the meta-analysis description, chromosomes with less than nine original grain weight QTL were analysed using the Goffinet and Gerber^23^ algorithm, which identified 36 MQTL across 11 chromosomes (except chromosome 3) with a 95% confidence interval and the lowest akaike information content AIC value (Table 1). The model that had a low AIC value while analysing was thought to indicate the importance of the MQTL discovered in this method. The chromosome 3 with 22 initial QTL was analysed using the Goffinet and Gerber algorithm as well as the Veyrieras approach resulted in identification of three MQTL with a 95 percent confidence interval. These three MQTL (MQTL3.1, MQTL3.2 and MQTL3.3) were declared true MQTL and considered for further analysis and validation, since they were identified using both methods with low AIC, corrected AIC (AICc and AIC3), Bayesian information criterion (BIC), and average weight of evidence (AWE) criteria. These three significant MQTL were located on chromosome 3at 39.5 cM, 92.82 cM, and 116.66 cM, respectively. These MQTL had incredibly small intervals, with MQTL3.1 having 0.49 Mb, MQTL3.2 having 0.63 Mb, and MQTL3.3 having just 0.08 Mb intervals (Fig. 4). The weights of these MQTL, on the other hand, were highly significant, with values 0.19, 0.33 and 0.37, respectively (Table 2). The fact that MQTL's physical lengths were so modest in comparison to their genetic lengths demonstrates the significance of these three MQTL (Supplementary Figure S1).

**Table 1:** Chromosome-wise list of initial QTL and identified MQTL.

Chromosome	Number of QTL	Number of MQTL	Model	Method
1	8	4	Model 4	Gerber and Goffinet
2	14	4	Model 4	Gerber and Goffinet
3	22	3	AIC, AICc, AIC3,BIC, AWE	Veyrieras
4	3	3	Model 3	Gerber and Goffinet
5	14	4	Model 4	Gerber and Goffinet
6	9	2	Model 2	Gerber and Goffinet
7	5	3	Model 3	Gerber and Goffinet
8	12	4	Model 4	Gerber and Goffinet
9	9	3	Model 3	Gerber and Goffinet
10	8	3	Model 3	Gerber and Goffinet
11	6	4	Model 4	Gerber and Goffinet
12	4	2	Model 2	Gerber and Goffinet
Total	114	39

**Table 2:** List and information on identified significant MQTL in the study.

Sl No	Meta-QTL	Chromosome	Positions	Start (cM)	End (cM)	Peak marker	Weight	CI (95%)	Interval (Mb)	Number of genes in the interval	Flanking markers
1	MQTL3.1	3	39.5	38.32	40.64	RM7197	0.19	2.33	0.49	73	RM14757-RM4321
2	MQTL3.2	3	92.82	91.54	94.13	RM15456	0.33	2.64	0.63	93	RM15442-RM15484
3	MQTL3.3	3	116.66	116.47	116.8	RM15832	0.37	0.36	0.08	13	RM15829-RM15834

### Mining genes underlying MQTL regions

For discovering the genes underlying MQTL regions, the relative physical positions of the closest flanking markers of MQTL were sought from the Gramene database. Genes within intervals of three MQTL on chromosome 3 were obtained from Rice MSU (http://rice.plantbiology.msu.edu/). Information on a total of 179 genes was extracted from intervals of identified MQTLs (Supplementary file 2). The MQTL3.1, having an interval of 0.49 Mb, accommodates a total of 73 genes, while MQTL3.2, with an interval of 0.63 Mb, accommodates 93 genes. The MQTL3.3 was found to have a very small interval of just 0.08 Mb containing 13 genes with a higher weight factor than the other two MQTL (Table 2). Even though 179 genes were found within the intervals of identified MQTL, only 25 genes were characterized for different biological functions (Table 3), including five genes controlling biological processes associated with grain weight and shape. Even though MQTL3.2 had the largest number of genes among the three MQTL, a greater number of characterized genes were found in MQTL3.1, followed by MQTL3.2, and a very small number were found in MQTL3.3. Among the characterized genes, four loci in the MQTL3.1 interval (*LOC*_*Os03g17690*, *LOC_Os03g17860*, *LOC_Os03g18050* and *LOC_Os03g18130*) and one locus in MQTL3.3 interval (*LOC_Os03g51330*) were found associated with grain development related functions. Conserved domain information for all the genes under three MQTL were used to understand the gene functions and found many ubiquitination pathway related genes, auxin biosynthesis genes, pectin methyl biosynthesis genes, and sucrose metabolism regulating genes in these regions (Supplementary file 3). Amino acid sequence BLAST of 25 characterized genes underlying three MQTL regions identified orthologue protein sequences in the other four core cereals. Complete syntenic relationship between rice and sorghum for all 25 amino acid sequences followed by maize and barley lacking synteny to amino acid sequences of *LOC_Os03g42290* and *LOC_Os03g18120*, respectively. However, wheat genome displayed lower synteny than the other three cereals (Supplementary file 4).

**Table 3:** List of characterized genes underlying identified significant MQTL.

Sl. No	MQTL	Locu ID	Position
1	MQTL3.1	LOC_Os03g17460	9,710,092—9,708,206
2	MQTL3.1	LOC_Os03g17470	9,714,748—9,712,370
3	MQTL3.1	LOC_Os03g17480	9,719,907—9,717,252
4	MQTL3.1	LOC_Os03g17570	9,768,656—9,759,479
5	MQTL3.1	LOC_Os03g17610	9,797,352—9,799,915
6	MQTL3.1	LOC_Os03g17690*	9,843,327—9,846,747
7	MQTL3.1	LOC_Os03g17700	9,850,473—9,847,700
8	MQTL3.1	LOC_Os03g17780	9,893,421—9,896,633
9	MQTL3.1	LOC_Os03g17790	9,900,400—9,899,758
10	MQTL3.1	LOC_Os03g17860*	9,955,138—9,952,788
11	MQTL3.1	LOC_Os03g17870	9,957,429—9,958,013
12	MQTL3.1	LOC_Os03g17980	9,961,503—9,960,708
13	MQTL3.1	LOC_Os03g18050*	10,057,658—10,058,281
14	MQTL3.1	LOC_Os03g18110	10,105,981—10,108,026
15	MQTL3.1	LOC_Os03g18120	10,116,219—10,120,924
16	MQTL3.1	LOC_Os03g18130*	10,124,384—10,119,873
17	MQTL3.1	LOC_Os03g18140	10,147,977—10,153,118
18	MQTL3.1	LOC_Os03g18150	10,163,441—10,165,477
19	MQTL3.2	LOC_Os03g41600	23,127,983—23,128,906
20	MQTL3.2	LOC_Os03g42020	23,341,805—23,347,331
21	MQTL3.2	LOC_Os03g42100	23,434,712—23,433,316
22	MQTL3.2	LOC_Os03g42110	23,442,337—23,438,157
23	MQTL3.2	LOC_Os03g42200	23,475,827—23,472,278
24	MQTL3.2	LOC_Os03g42290	23,533,463—23,538,865
25	MQTL3.3	LOC_Os03g51330*	29,370,719—29,373,265

*genes directly involved in grain weight or grain shape related biological processes.

### Ontology of genes within the MQTL regions and expression analysis of candidate genes

**Figure 5:**
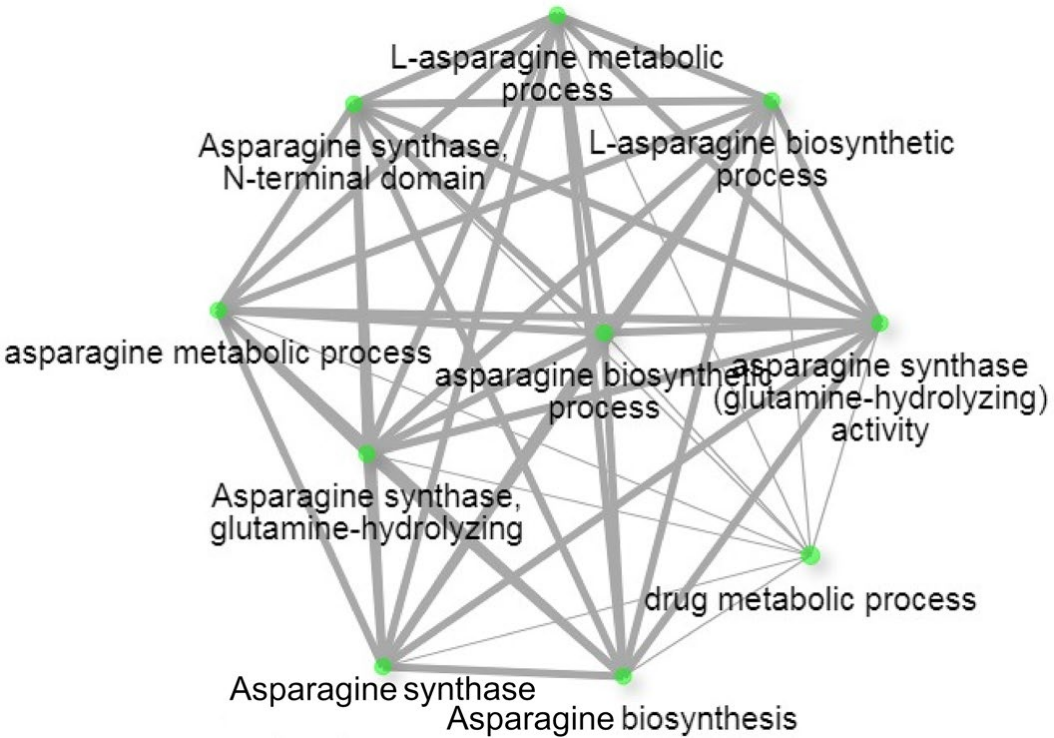
Network display of five grain related genes underlying identified MQTL involved in asparagine biosynthesis and metabolism pathways having significant role in regulating source to sink relation in rice. The green nodes in the network indicate different asparagine biosynthesis and metabolic pathways and grey connecting lines indicate interaction between the processes of pathways to contribute towards establishing source to sink relationship.

**Figure 6:**
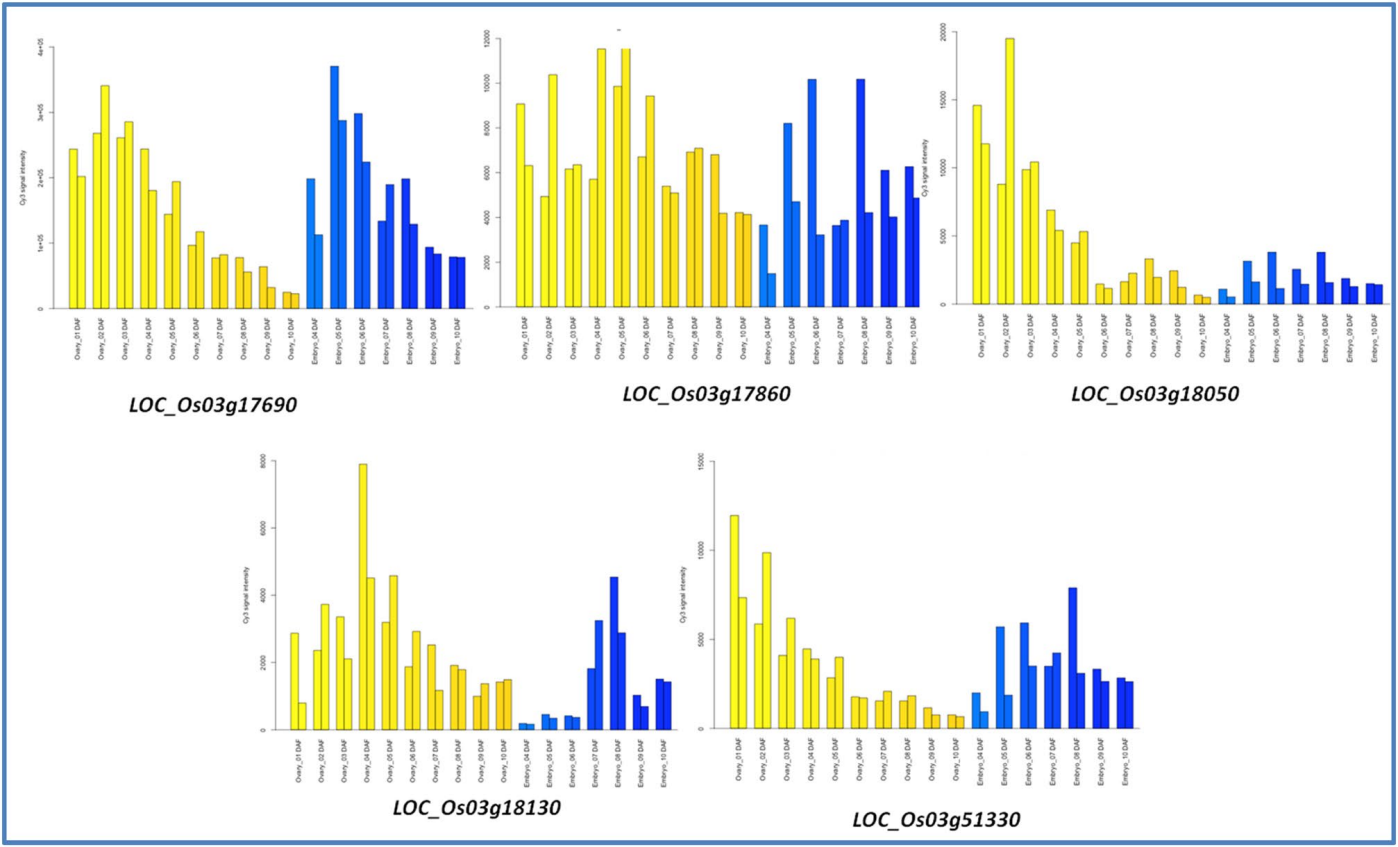
Graphical representation of expression of candidate genes at early grain development stage. The yellow bars taken in two replicates indicate expression of candidate genes in ovary at different days after flowering, whereas the blue bars indicate expression of genes in embryo at different days after flowering, and represented in two replications.

The GO analysis of genes within MQTL regions was performed by taking genes in individual MQTL. Gene ontology analysis within the MQTL3.1 and MQTL3.3 regions was unsuccessful due to low gene numbers and gene ontology terms. However, MQTL3.2, with a 0.63 Mb interval containing 93 genes, was found to have sufficient GO terms to map. Ontology annotation for these genes predicted the involvement of genes in many biological processes such as biological regulation, metabolic process, macromolecule synthetic pathways, transcription, gene expression, etc., indicating direct or indirect association of these genes in grain development (Supplementary Figure S2). Further, the five genes identified as directly related to grain development and considered as candidate genes (Table 4) were analyzed separately for functional annotation using the ShinyGO v0.741 (http://bioinformatics.sdstate.edu/go/) online tool. These genes were found to be associated with many asparagine metabolism related pathways (Fig. 5). These genes play a significant role in understanding the source-sink relationship in rice^24^. Micro-array based expression analysis datasets from RiceXPro revealed changes in the expression patterns of five candidate genes studied. Among five candidate genes, locus *LOC_Os03g17860* (*PDIL* gene) was found to have continuous expression from early stages of ovary and embryo development to later stages of maturation (Fig. 6). Locus *LOC_Os03g18050* (*SAUR* gene) was found to up regulate early ovary development followed by down regulation during embryo development. Other three candidate genes were found to have significant up regulation during early stages of ovary and embryo development.

**Table 4:** Details of candidate genes identified under MQTL regions associated with grain development.

MQTL	Locus ID	MSU ID	GO term	Gene description
MQTL3.1	Os03g0285700	LOC_Os03g17690	Metabolic process	Similar to L-ascorbate peroxidase
MQTL3.1	Os03g0287900	LOC_Os03g17860	Cellular homeostasis	Similar to Protein disulfide isomerase
MQTL3.1	Os03g0290300	LOC_Os03g18050	Molecular function	Auxin responsive protein
MQTL3.1	Os03g0291500	LOC_Os03g18130	Biosynthetic process	Asparagine synthase domain containing protein
MQTL3.3	Os03g0723000	LOC_Os03g51330	Cellular component	GRAS transcription factor domain containing protein

### Validation of MQTL

**Figure 7:**
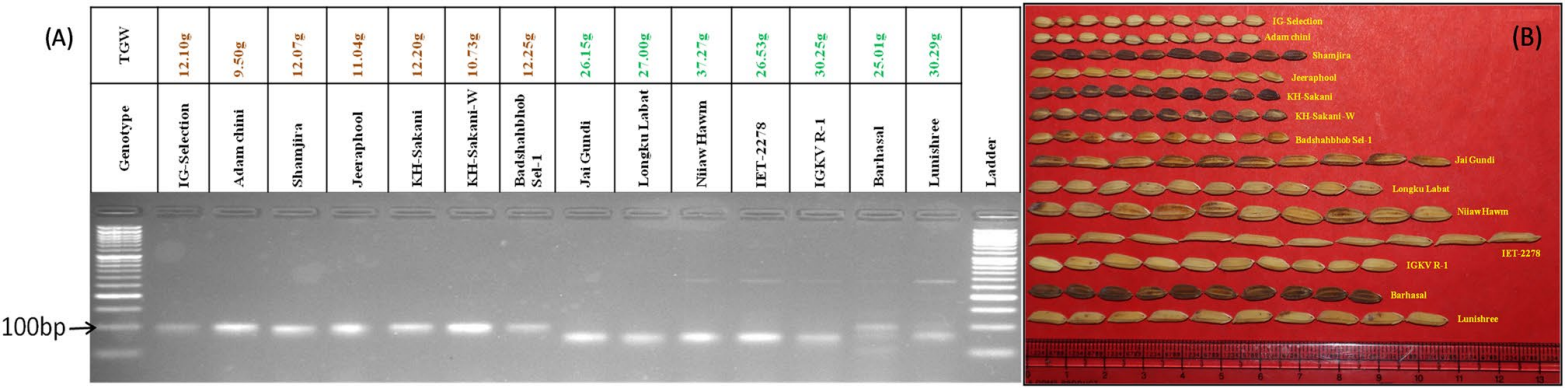
(**A**) Validation of marker RM7197, peak marker of MQTL3.1 on set of seven low grain weight and seven high grain weight genotypes. The allele with 100 bp linked to low grain weight and 85 bp allele linked to high grain weight. (**B**) Representation of grain size and shape of genotypes used for validation of identified MQTL.

Considering the consensus map as reference, three peak markers present in the middle of the identified MQTL region were opted for validation of MQTL. Markers RM7197, RM15456, and RM15832 from MQTL3.1, MQTL3.2, and MQTL3.3, respectively, were used for validation of these MQTL. Out of three selected markers, only RM7197 could clearly differentiate the genotypes into two phenotypic classes, i.e., high and low grain weight. Hence, MQTL3.1 was validated beyond doubt by RM7197. The allele with an amplicon size of 100 bp was found specifically associated with low grain weight genotypes while the alternate allele with an amplicon size of 85 bp was specific to higher grain weight (Fig. 7). The validated SSR marker RM7197 is located at 9,888,524 bp on rice chromosome three between identified candidate genes LOC_Os03g17690 and LOC_Os03g17860. Even though the marker sequence was found within the inter-genic region, it was found to surrogate all four candidates in the MQTL3.1 region. However, the other two MQTL were not clearly validated in the study, indicating the need for the identification of more appropriate markers for further validation of those MQTL.

**Figure 8:**
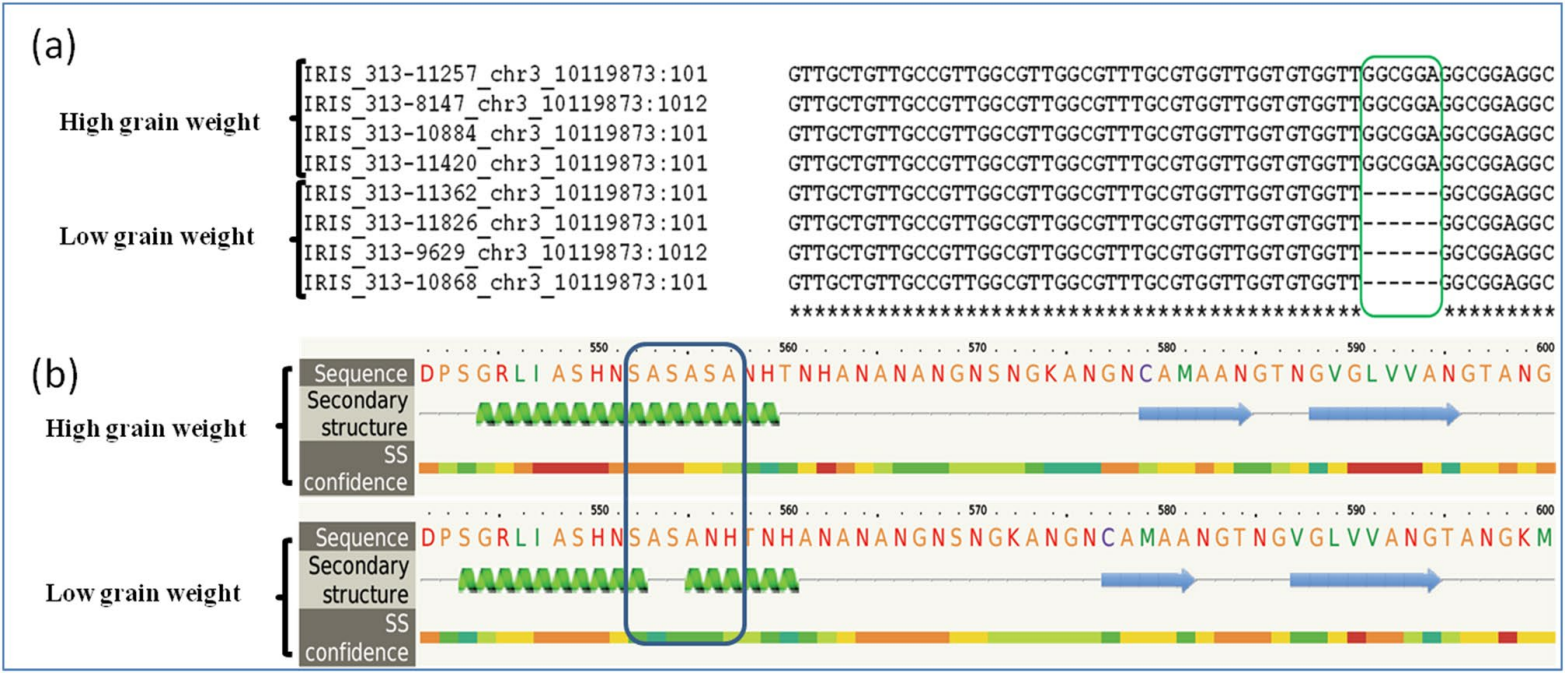
Sequence variation between low and high grain weight genotypes for *LOC_Os03g18130* under MQTL3.1 retrieved from 3 k rice genome project. (**a**) A six base pair deletion in the last exon of the gene in low grain weight genotypes and (**b**) two amino acids, alanine and serine deleted in amino acid coding sequence which led to interruption of secondary structure (α-helix) of asparagine synthetase b protein in low grain weight genotypes.

The gene sequence differences between eight genotypes from the 3 K rice genome project (Supplementary file 6) revealed sequence variations between genotypes for five candidate genes identified under two MQTL. However, differences in sequences for loci LOC Os03g17690, LOC Os03g17860, and LOC Os03g18050 under MQTL3.1, and locus LOC Os03g51330 under MQTL3.3 did not distinguish low grain weight and high grain weight genotype classes. Interestingly, the gene sequence of eight genotypes at locus LOC_Os03g18130 under MQTL3.1 exhibited prominent sequence differences for genotypes of different grain weight classes. Three single nucleotide changes distinguished genotype classes at 317 bp, 349 bp, and 395 bp, followed by two nucleotide deletions in high grain weight genotypes, were noticed at 2037 bp from 5’ end. Unfortunately, these SNPs and two nucleotide differences were found in the intronic regions of the gene and their contribution to the grain weight difference was ruled out. A six nucleotide deletion at 3646 bp in low grain weight genotypes was observed, which is located on the last exon of the gene (Fig. 8a). The deleted nucleotides code for alanine and serine amino acids in high grain weight genotypes. The deletion of two amino acids interrupted the α-helix in the secondary structure of the asparagine synthetase B protein (Fig. 8b), which also made minimal changes to the 3D structure (Supplementary Figure S3). Since the deletion was found in the coding region, this sequence change is expected to have an impact on gene expression.

## Discussion

Understanding the genetic architecture of complex quantitative traits is a prelude to crop improvement. Grain weight in rice, being a metric trait, has a significant impact on grain yield, and thus needs a thorough dissection and deployment in the rice yield improvement breeding programme. Several researchers over the last couple of decades have characterised and mapped several genetic loci responsible for inheritance of rice grain weight^25–28^. However, many of these findings done on different bi-parental populations often resulted in higher confidence interval that limited their validation and utilization in practical plant breeding^29,30^. The information on QTL identified in independent experiments over various populations across diverse environments can be integrated to identify true and consistent QTL through a meta-analysis approach^23^. Few studies have located MQTL^14,19,31^ to mine candidate genes.

In the present study, meta-QTL analysis was conducted specifically for thousand grain weight in rice. For comprehensive identification of consistent QTL responsible for grain weight in rice, information from 22 independent investigations identifying 114 QTL was used (Supplementary file 1). The initial QTL identified were spread across 12 rice chromosomes, indicating metric inheritance of the trait with several causative alleles over all the chromosomes (Fig. 2). A similar trend of QTL distribution on all chromosomes was reported by Islam et al.^32^ for salt tolerance in rice. Even though 39 MQTL were identified from 114 original QTL, only three MQTL on chromosome 3 were considered significant based on AIC information (Table 1). The presence of a greater number of initial QTL for grain weight on chromosome 3 hinted at the chances of identification of a consistent MQTL (Fig. 3A). Further, earlier researchers like Xu et al.^33^ and Zhu et al.^34^ identified a few major QTL on chromosome 3 for grain weight. Three MQTL identified on chromosome 3 were found to have very low interval and comparatively high weight (Table 2), which is advantageous for utilization in strategic breeding programs^35^. The genome-wide association studies carried out on two diverse rice panels did not identify any QTL either for grain weight^36,37^ or for grain yield^38^ on chromosome three owing to population size and marker density used in these studies. Consensus projection of all the minor and major QTL identified in different studies into a single or few MQTL, which explains the variation of all included QTL, signifies the power of meta-QTL identification. Hence, identified consistent MQTL with short interval have potential use in marker assisted backcross breeding programs for rapid elimination of linkage drag. The identified MQTL regions were found rich in gene content, harboring a total of 179 gene loci within their intervals. Several of these genes were not annotated, and only 25 of them, were to be found characterized and annotated. These included important genes like *GSTL3* (*LOC*_*Os03g17460*) and *GSTL1* (*LOC_Os03g17480*) responsible for stress tolerance^39,40^, while *PRR73* (*LOC_Os03g17570*) regulates salt tolerance^41^ in rice. However, these characterized genes are not related to grain weight and are instead associated with some important biological processes. The Gene Ontology survey helped understand the functions of genes within MQTL. The gene ontology plot from the MQTL3.2 region depicted the roles of loci in different biological and metabolic processes (Supplementary Figure S2). These genes are associated with many physiological and molecular pathways related to grain development^42^. The group of genes significantly associated with GO terms from MQTL regions might enrich phenotype expression of grain weight and aid in selection. Further, a sequence blast to identify conserved domains for all 179 genes unraveled the information on important genes and their functions, which contribute towards the biological process of grain development. Genes responsible for the ubiquitination pathway, auxin biosynthesis pathway, pectin methyl transferase coding genes and sucrose metabolism pathway regulating genes were found within the identified MQTL regions. These pathways have significant roles to contribute for early grain development, including cell division in endosperm^43^ and grain maturation by sink organ sucrose metabolism^44,45^. The comparison of orthologue amino acid sequences of other closely related cereals revealed that these proteins are conserved across species and are responsible for important biological functions, leading to plant development as similar to rice. The availability of orthologue regions in closely related cereals increases the significance of identified MQTL and also verifies their stability^46,47^. The orthologue sequence search also included the candidate genes identified in this study, the protein expressions of amino acid sequences in other cereals were found to be similar to those in the rice plant. Thus, that verifies the stability of identified candidate genes in this study.

Four loci from MQTL3.1 and one from MQTL3.3 were reported to have associations with grain development related processes. Locus *LOC_Os03g17690* found in MQTL3.1 was associated with *OsAPX1* gene plays significant role in embryogenesis^48^ and also in heat stress tolerance^49^. The Gene *PDIL* (*LOC_Os03g17860*) was responsible for accumulation of seed storage^50^, gene *SAUR* (*LOC_Os03g18050*) was influencing reproductive development^51^ while *OsASN1* (*LOC_Os03g18130*) regulating asparagine dependent rice development^52^. These functions of identified gene loci within MQTL3.1 define its importance in the improvement of rice grain weight. None of the genes identified within the MQTL3.2 region were found directly associated with grain weight. However, their importance in other biological processes leading to grain yield was found to be significant. Among the genes within MQTL3.3, gene *OsGRAS19* (*LOC_Os03g51330*) was reported to regulate grain shape in rice^53^. Apart from directly influencing grain development processes, these genes were found to be associated with asparagine biosynthesis and asparagine metabolic processes (Fig. 5). It is reported that asparagine along with glutamine regulates source to sink relations in rice plant^52^. Glutamine and asparagine are the two important forms of N transport from root to shoot in rice plants. Further, reproductive organ specific expression of identified candidate genes under defined MQTL regions indicated the role of early embryo development stages for enhancing grain weight. Utilization of these MQTL in plant breeding enhances the chance of improving source to sink relations, thereby increasing grain weight and yield in rice.

Among three random rice markers used for validation of MQTL on a set of extreme genotypes consisting of seven low grain weights and seven high grain weights, only one marker (RM7197) clearly differentiated two extreme categories (Fig. 7). Segregation of marker amplicons with low and high seed weight classes of genotypes validates their linkage with the respective MQTL. The validated marker was the peak marker of MQTL3.1, indicating the marker was tightly linked to MQTL3.1. The validated marker, RM7197, has scope in practical marker aided plant breeding to incorporate the MQTL3.1 region into an elite background to enhance grain weight. The association of the RM7197 marker with MQTL3.1 indicates its association with all other genes underlying MQTL3.1. Hence, it can be used as a surrogate to track them in marker-assisted rice breeding programs for improving grain weight. The remaining two markers related to the remaining two MQTL were not clearly validated owing to very few QTL/genes involved in the MQTL formation. In MQTL3.2, only one candidate gene and a few minor QTL were reported in the region; whereas, within MQTL3.3, no candidate genes were identified and only two minor QTL were present. Therefore, the marker linked to very small numbers of alleles responsible for grain weight might not be possible to associate with a complete phenotype of grain weight in genotypes with extreme phenotypes. Hence, there was a lack of association with phenotype in the validation.

The six nucleotide deletion on the last exon of the gene was noticed in the gene sequence of low grain weight genotypes. The deleted sequence 5’GGCGGA3’ with the complementary sequence 5’TCCGCC3’ in high grain weight genotypes codes for two amino acids, serine and alanine, which play a significant role in aspargine biosynthesis^54^. The efficiency of serine: glyoxylate aminotransferase or alanine: glyoxylate aminotransferase (AGT1) activity in the asparagine biosynthetic pathway significantly increases when glycolate is an amino acceptor and serine or alanine as an amino donor^55^. The deletion of coding sequences of these amino acids negatively impacts the asparagine biosynthetic pathway, which indirectly reduces the strength of the sink-to-source relationship. Hence, this change in gene sequence may be one of the genetic causes of the change in grain weight. This evidence from gene sequence information of genotypes from the 3 K rice genome project reiterated the importance of identified MQTL for grain weight.

## Conclusion

By collating the results of independent investigations conducted over various environments and identification of true consensus genomic region for grain weight paves new avenue for utilization in practical rice breeding. In this study, three significant and consistent consensus genomic regions for grain weight were identified in rice through a QTL-meta analysis approach. The intervals of these MQTL contains many candidate genes responsible for asparagine metabolic pathway and GRAS transcription factor domains which regulates source to sink relationship and grain weight in rice. This provides insight into pathways underlying grain weight character and genes responsible for variable grain weight in rice. The marker RM7197 validated for identified meta-QTL has significant scope in incorporating all the genes underlying MQTL3.1 for improving grain weight in rice. The outcome of this investigation has significant application in practical rice breeding programs to incorporate these MQTL for grain weight improvement through marker-aided breeding programs.

## Materials and methods

### Bibliographic survey and data generation

A comprehensive survey of literature related to QTL mapping for grain weight in rice published over the last two decades was performed to aggregate the information (Supplementary file 1). The information on the number and type of molecular markers, genetic map, type and size of mapping populations, parents used, LOD scores, genetic distances in linkage groups and proportion of phenotypic variance explained (R^2^ values) were collated from published literature. Information on a total of 114 QTL mapped exclusively for grain weight over environments across populations along with their genetic maps were collected, tabulated, and summarized. A few studies with incomplete information on genetic maps were excluded from the study. The population size of progenies in the mapping populations of various studies varied from 39 to 353 progenies evaluated over different locations encompassing different studies. Separate input text files for map and QTL information were created as per the requirements of Biomercator v4.2^17^. This information was considered as primary information for the present investigation.

### Consensus map development and QTL projection

Forehand to MQTL analysis, a consensus genetic map was developed based on collated information of genetic maps from published studies using Biomercator v4.2^16,17^. The genetic map well saturated with 19,180 SSR markers available in the Gramene database (https://archive.gramene.org/markers/) was used as the reference genetic map for developing the consensus map. The information on individual map files for 114 grain weight specific QTL (original/initial QTL) was integrated on to the consensus map for QTL projection. In order to incorporate information from the SNP markers linked to original QTL, the positions of SNP markers in the rice genome were determined and the closest SSR markers corresponding to them were chosen for projecting QTL on the consensus map as suggested by Khahani et al.^56^. The QTL projection on the consensus map was based on R^2^ explained by QTL, LOD score, QTL positions, and confidence intervals of initial QTL. For the original QTL, the confidence interval (CI) used to project them on the consensus map was calculated following the Darvasi and Soller^57^ equation: $\frac{530}{{NR}^{2}}$, in which N is the size of the population and R^2^ is the phenotypic variation explained by the QTL. The QTL position on the consensus map was projected by considering the marker interval of the QTL on the original map and the corresponding position on the consensus map. A Gaussian distribution rule was used to calculate the new CI on the consensus map as suggested by Veyrieras et al.^16^.

### QTL meta-analysis

A consensus genetic map of each chromosome with projected QTL for grain weight was subjected to MQTL analysis following the default parameter setting in BioMercator v4.2 (https://urgi.versailles.inra.fr/Tools/BioMercator-V4)^16,17,58^. The method of analysis was selected based on initial QTL numbers on each chromosome; if a chromosome contains more than nine QTL, the ‘Veyrieras’ method or otherwise the ‘Gerber and Goffinet’ method was followed. An appropriate model for the identification of MQTL was chosen based on Akaike Information Criterion (AIC), corrected Akaike Information Criterion (AICc and AIC3), Bayesian Information Criterion (BIC) and/or Average Weight of Evidence (AWE) criteria^16^. The lowest number of models predicted by at least three of these criteria was considered the best. The MQTL identified by the best model based on the lowest value for the aforesaid criteria was reported^59^.

### Discovery of genes within Meta-QTL regions

Based on the results of MQTL analysis, the anchor positions of significant MQTL were identified by considering flanking marker positions obtained from the Gramene database. For the process called mapping, physical positions of flanking markers were used as inputs in the search field of Rice MSU (http://rice.plantbiology.msu.edu/) to discover and extract information on the number of genes, locus IDs, and annotated data related to MQTL regions. For each loci identified under MQTL region, the information on conserved domain and gene function was extracted from Rice MSU (http://rice.plantbiology.msu.edu/) and NCBI-conserved domain database (https://www.ncbi.nlm.nih.gov/Structure/cdd/wrpsb.cgi). Further, amino acid sequences of characterized genes under identified MQTL regions were used to find orthologues in other core cereals using the NCBI orthologue search database (https://www.ncbi.nlm.nih.gov/). The locus IDs of genes from the MQTL regions were used as input for the gene ontology (GO) search.

### Gene ontology analysis and expression analysis

Selecting appropriate gene ontology terms from the list of mapped genes in a specific genomic region is termed as GO annotation. The information on locus ID of genes discovered in the MQTL region was functionally classified following the single enrichment analysis (SEA) tool in the web-based AgriGOv2.0 (http://systemsbiology.cau.edu.cn/agriGOv2/) interface. The necessary parameters like Benjamini-Yekutieli (FDR under dependency) under multiple adjustment test and significance at 5% were set before analysis^32^. The GO terms include the involvement of genes in biological processes, cellular processes, metabolic processes and regulation, etc. Among these genes, grain development related genes were identified based on available literature and were considered as candidate genes for grain weight. The expression data of these genes were downloaded from RiceXPro database. RXP_0011 dataset, which contains micro-array based expression data of grain early stage development, was considered for further interpretation.

### Validation of identified MQTL by linked markers

In traditional QTL validation, it is important to develop near isogenic or transgenic lines for assessing the effect of identified QTL. Validation of MQTL conceptually differs from that of QTL validation owing to its property of inclusive QTL^32^. According to the algorithmic definition of meta-analysis, MQTL identification in a condensed genomic region assures the validation of known QTL in the region^23^. Hence, the identification of a linked surrogate marker and validating it on a set of genotypes with extreme phenotypes is practically significant. Significant association of marker alleles with phenotypes ensures the presence of at least a few alleles of QTL present in the MQTL. Several researchers validated identified MQTL following the aforesaid approach^14,32,60^. In the present study, MQTL associated peak-SSR markers from each MQTL were opted for validation on a set of 14 genotypes, comprising seven genotypes each from extreme low and high grain weight categories available at ICAR-National Rice Research Institute Cuttack. The experimental genotypes were selected from a panel of 300 genotypes evaluated over the last five years, and a mean of thousand grain weight data was considered. The genotypes with less than 12.50 g were considered as low grain weight set and those with more than 25.00 g were considered as high grain weight set. Among selected genotypes, Niiaw Hawm (37.27 g) the recorded highest grain weight, while Adam Chini (9.50 g) recorded the lowest grain weight (Supplementary file 5). Seedlings were grown in pots at laboratory conditions and genomic DNA was extracted using the CTAB method^61^. The quality and quantity of genomic DNA were tested before thermal amplification of regions corresponding to MQTL-linked markers. Amplified fragments were visualized using gel electrophoresis and a documentation system.

Further, the gene sequences of four loci under MQTL3.1 and one locus under MQTL3.3 for a set of eight genotypes, four low grain weight and four high grain weight^62^ in the 3 K rice genome project were retrieved from the rice functional genomics and breeding (RFGB) database (https://www.rmbreeding.cn/public/searchbak). The indica accessions of Indian origin were selected to understand the gene sequence differences between low grain weight and high grain weight genotypes (Supplementary file 6). The gene sequences of eight genotypes were subjected to multiple sequence alignment using the **MU**ltiple **S**equence **C**omparison by **L**og- **E**xpectation (MUSCLE) tool (https://www.ebi.ac.uk/Tools/msa/muscle/). Upon identification of sequence differences between genotypes, their position on the gene sequence and functional contributions to variation were explored. The protein structure variation due to sequence differences was analyzed using the Phyre2 webportal (http://www.sbg.bio.ic.ac.uk/~phyre2/html/page.cgi?id=index)^63^ and represented in for visualization.

## Supplementary Information

Supplementary Information 1.

Supplementary Information 2.

Supplementary Information 3.

Supplementary Information 4.

Supplementary Information 5.

Supplementary Information 6.

Supplementary Information 7.

Supplementary Information 8.

Supplementary Information 9.

## Data Availability

All data included in this study are available upon request by contact with the corresponding author. The data used for gene expression analysis was retrieved from RXP_0011 data set in RiceXPro database (https://ricexpro.dna.affrc.go.jp/Zapping/.) and sequence information for eight genotypes was retrieved from rice functional genomics and breeding database (https://www.rmbreeding.cn/).
