# Supplementary figures and images for: Understanding complex genetic architecture of rice grain weight through QTL-meta analysis and candidate gene identification
Source: Sci Rep. 2022 Aug 16;12:13832. doi: 10.1038/s41598-022-17402-w (PMC9381546; doi:10.1038/s41598-022-17402-w)

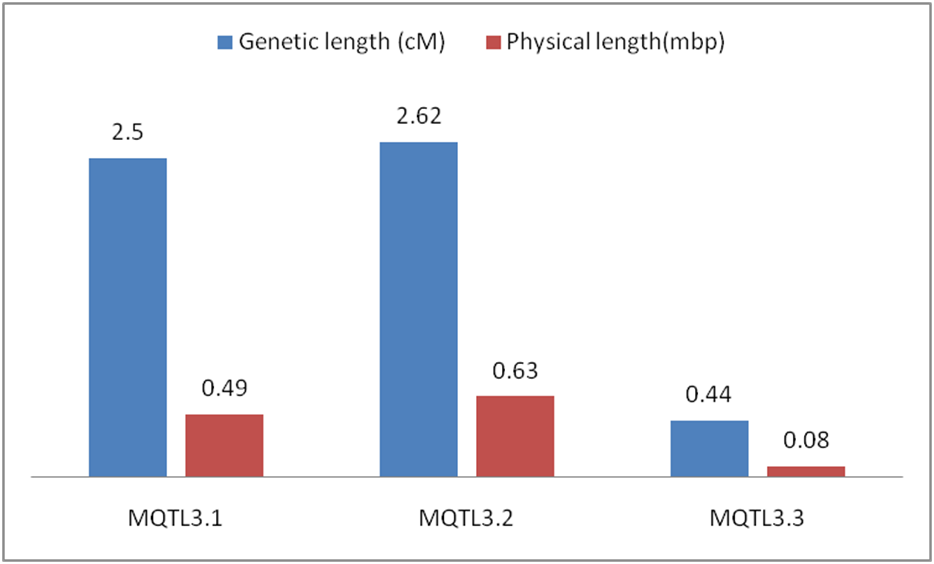


Supplementary Figure S1. Comparison of physical and genetic length of identified MQTL.

Supplement: Supplementary file 1 — Supplementary Information 1. [file 41598_2022_17402_MOESM1_ESM.docx]
