# Supplementary material for: Understanding complex genetic architecture of rice grain weight through QTL-meta analysis and candidate gene identification
Source: Sci Rep. 2022 Aug 16;12:13832. doi: 10.1038/s41598-022-17402-w (PMC9381546; doi:10.1038/s41598-022-17402-w)
Supplement: Supplementary file 2 — Supplementary Information 2. [file 41598_2022_17402_MOESM2_ESM.docx]

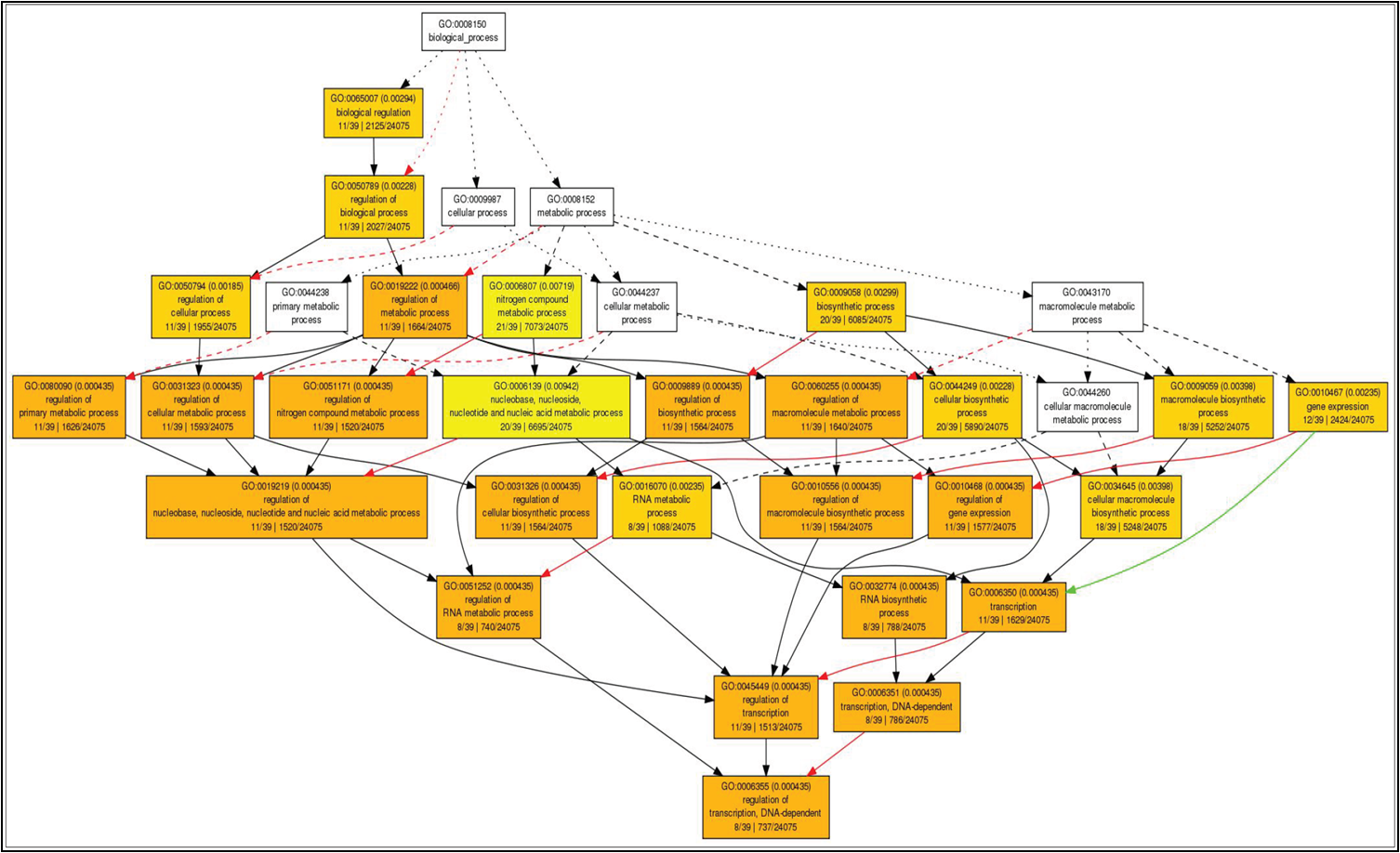


Supplementary Figure S2: Gene ontology of genes under MQTL3.2. Solid black arrow indicate direct involvement of gene in the process, Solid yellow arrow indicates partial involvement, solid red arrow indicate positive regulation, solid green arrow indicate negative regulation of the process, dashed black arrow indicate two significant nodes of action of gene and dotted black arrow indicate single significant node for regulation of process. The color intensity of GO terms indicates level of their significance.
