# Supplementary material for: Understanding complex genetic architecture of rice grain weight through QTL-meta analysis and candidate gene identification
Source: Sci Rep. 2022 Aug 16;12:13832. doi: 10.1038/s41598-022-17402-w (PMC9381546; doi:10.1038/s41598-022-17402-w)
Supplement: Supplementary file 3 — Supplementary Information 3. [file 41598_2022_17402_MOESM3_ESM.docx]

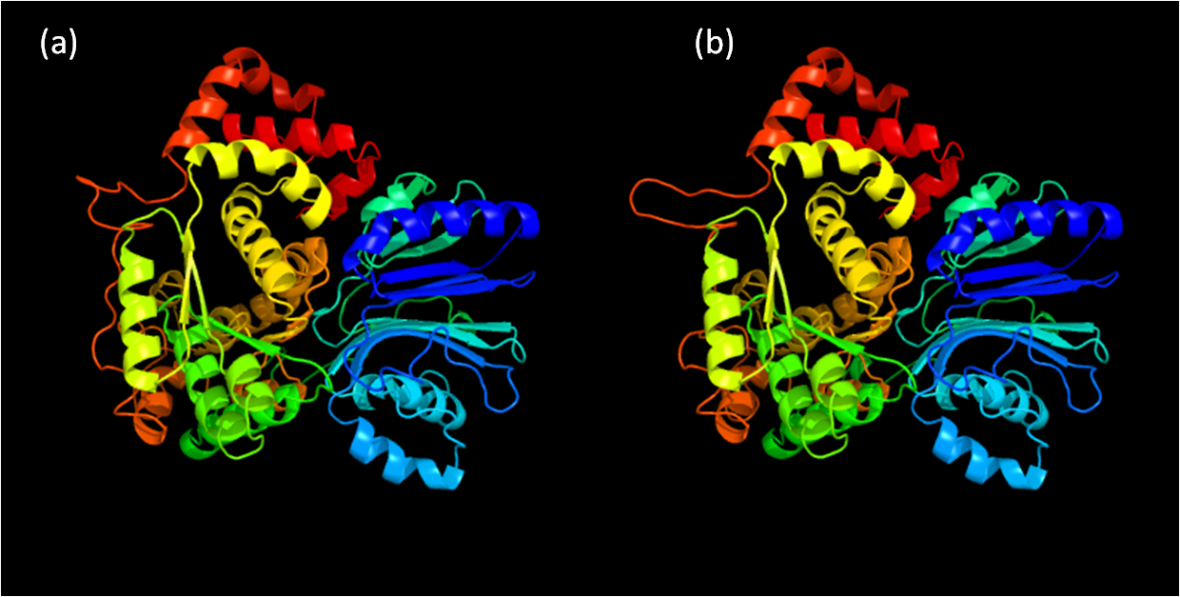


Supplementary Figure S3. Comparison of 3D structure of asparagines synthetase enzyme coded by LOC_Os03g18130 gene obtained from (a) low grain weight and (b) high grain weight genotypes. Low grain weight accession names: ARC 11571, KEYA NUNIA, JC157 and HIRA KHI. High grain weight accession names: ARC 12757, T 757, ARC 12493 and PISINI (details of the accessions are provided in supplementary file 6).
