# Supplementary material for: Understanding complex genetic architecture of rice grain weight through QTL-meta analysis and candidate gene identification
Source: Sci Rep. 2022 Aug 16;12:13832. doi: 10.1038/s41598-022-17402-w (PMC9381546; doi:10.1038/s41598-022-17402-w)
Supplement: Supplementary file 4 — Supplementary Information 4. [file 41598_2022_17402_MOESM4_ESM.docx]

| Supplementary file 1: Primary information on original QTL used for the present study | | | | | | |
| --- | --- | --- | --- | --- | --- | --- |
| Sl. No. | Title | Parents | Population | Population size | Number of QTLs | Reference |
| 1 | Dissection of three quantitative trait loci for grain size on the long arm of chromosome 10 in rice (*Oryza sativa* L.) | Teqing x IRBB52 | RIL | 203 | 3 | Zhu et al. (2019) |
| 2 | Dissection of the qTGW1.1 region into two tightly-linked minor QTLs having stable effects for grain weight in rice | ZS97 x MY46 | BIL | 237 | 2 | Zhang et al. (2016) |
| 3 | Fine-mapping of qTGW2, a quantitative trait locus for grain weight in rice (Oryza sativa L.) | Teqing x IRBB52 | BIL | 250 | 4 | Zhang et al. (2020) |
| 4 | Combined Linkage Mapping and Genome-Wide Association Study Identified QTLs Associated with Grain Shape and Weight in Rice (*Oryza sativa* L.) | cv. 93-11 x cv. Milyang352 | DH | 117 | 10 | Kang et al. (2020) |
| 5 | Validation of a QTL for Grain Size and Weight Using an Introgression Line from a Cross between *Oryza* *sativa* and *Oryza* *minuta* | IL188 X Nipponbare | SF2 | 166 | 2 | Feng et al. (2021) |
| 6 | QTL mapping of grain appearance quality traits and grain weight using a recombinant inbred population in rice (*Oryza sativa* L.). | G46B x K1075 | RIL | 182 | 2 | Gao et al. (2016) |
| 7 | QTL mapping of grain weight in rice and the validation of the QTL qTGW3.2 | D50 x HB277 | SF2 | 116 | 7 | Tang et al. (2013) |
| 8 | Mapping quantitative trait loci for yield, yield components and morphological traits in an advanced backcross population between *Oryza rufipogon* and the *Oryza sativa* cultivar Jefferson | Jefferson x IRGC 105491 | BC | 353 | 8 | Thomson et al. (2003) |
| 9 | QTL mapping and correlation analysis for 1000-grain weight and percentage of grains with chalkiness in rice | Sasanishiki x Habataki | BC | 39 | 9 | Bian et al. (2013) |
| 10 | Rapid Identification of Major QTLs Associated with Rice Grain Weight and Their Utilization | JY293 x M201 | SF2 | 234 | 2 | Xu et al. (2015) |
| 11 | An Efficient Strategy Combining SSR Markers- and Advanced QTL-seq-driven QTL Mapping Unravels Candidate Genes Regulating Grain Weight in Rice | IR64 x Sonasal | SF2 | 190 | 6 | Daware et al. (2016) |
| 12 | Fine mapping of qTGW10-20.8, a QTL having important contribution to grain weight variation in rice | Teqing x IRBB lines | RIL | 204 | 31 | Zhu et al. (2019) |
| 13 | Genetic Mapping of QTLs that Control Grain Characteristics in Rice (*Oryza sativa* L.) | Cheongcheong x Nagdong | DH | 120 | 1 | Wacera et al. (2015) |
| 14 | A Kelch Motif-Containing Serine/Threonine Protein Phosphatase Determines the Large Grain QTL Trait in Rice | CW23 x PA64 | SF2 | 178 | 4 | HU et al. (2012) |
| 15 | Identify QTLs for grain size and weight in common wild rice using chromosome segment substitution lines across six environments | CWR274 x 9311 | CSSL | 133 | 2 | Qi et al. (2017) |
| 16 | Mapping and validation of quantitative trait loci for spikelets per panicle and 1,000-grain weight in rice (*Oryza sativa* L.) | Teqing x Minghui | RIL | 63 | 4 | Liu et al. (2010) |
| 17 | Identification of Quantitative Trait Loci for Grain Traits in Japonica Rice | DL115 x XL005 | SF2 | 200 | 3 | Li et al. (2009) |
| 18 | Identification of Quantitative Trait Loci Associated with Grain Shape Using Cheongchenong/Nagdong Double Haploid Lines in Rice | Cheongcheong x Nagdong | DH | 120 | 3 | Wacera et al. (2016) |
| 19 | Molecular dissection of the genetic relationships of source, sink and transport tissue with yield traits in rice | Zhenshan 97 x Minghui 63 | RIL | 241 | 1 | Cui et al. (2003) |
| 20 | Identification of Genetic Overlaps for Salt and Drought Tolerance Using Simple Sequence Repeat Markers on an Advanced Backcross Population in Rice | Teqing × Binam | IL | 77 | 3 | Yun et al. (2012) |
| 21 | Background-Independent Quantitative Trait Loci  for Drought Tolerance Identified Using Advanced  Backcross Introgression Lines in Rice | IR64 × Tarom | BIL | 85 | 4 | Wang et al. (2013) |
| 22 |  | Teqing × Tarom | BIL | 72 | 3 |  |

**References**

Bian JM, Shi H, Li CJ, Zhu CL, Yu QY, Peng XS, Fu JR, He XP, Chen XR, Hu LF, Ouyang LJ. QTL mapping and correlation analysis for 1000-grain weight and percentage of grains with chalkiness in rice. Journal of genetics. 2013 Aug 1;92(2):281-7.

Cui K, Peng S, Xing Y, Yu S, Xu C, Zhang Q. Molecular dissection of the genetic relationships of source, sink and transport tissue with yield traits in rice. Theoretical and Applied Genetics. 2003 Feb;106(4):649-58.

Daware A, Das S, Srivastava R, Badoni S, Singh AK, Agarwal P, Parida SK, Tyagi AK. An efficient strategy combining SSR markers-and advanced QTL-seq-driven QTL mapping unravels candidate genes regulating grain weight in rice. Frontiers in plant science. 2016 Oct 26;7:1535.

Feng Y, Yuan X, Wang Y, Yang Y, Zhang M, Yu H, Xu Q, Wang S, Niu X. Validation of a QTL for Grain Size and Weight Using an Introgression Line from a Cross between Oryza sativa and Oryza minuta. Rice. 2021 Dec;14(1):1-2.

GAO FY, ZENG LH, Ling QI, LU XJ, REN JS, WU XT, SU XW, GAO YM, REN GJ. QTL mapping of grain appearance quality traits and grain weight using a recombinant inbred population in rice (Oryza sativa L.). Journal of integrative agriculture. 2016 Aug 1;15(8):1693-702.

Hu Z, He H, Zhang S, Sun F, Xin X, Wang W, Qian X, Yang J, Luo X. A Kelch motif‐containing serine/threonine protein phosphatase determines the large grain QTL trait in rice. Journal of integrative plant biology. 2012 Dec;54(12):979-90.

Kang JW, Kabange NR, Phyo Z, Park SY, Lee SM, Lee JY, Shin D, Cho JH, Park DS, Ko JM, Lee JH. Combined Linkage Mapping and Genome-Wide Association Study Identified QTLs Associated with Grain Shape and Weight in Rice (Oryza sativa L.). Agronomy. 2020 Oct;10(10):1532.

Li M, Xu L, Ren J, Cao G, Yu L, He H, Han L, Koh H. Identification of quantitative trait loci for grain traits in japonica rice. Scientia Agricultura Sinica. 2009;42(7):2255-61.

Liu T, Shao D, Kovi MR, Xing Y. Mapping and validation of quantitative trait loci for spikelets per panicle and 1,000-grain weight in rice (Oryza sativa L.). Theoretical and applied genetics. 2010 Mar 1;120(5):933-42.

Qi L, Sun Y, Li J, Su L, Zheng X, Wang X, Li K, Yang Q, Qiao W. Identify QTLs for grain size and weight in common wild rice using chromosome segment substitution lines across six environments. Breeding science. 2017:16082.

Tang SQ, Shao GN, Wei XJ, Chen ML, Sheng ZH, Luo J, Jiao GA, Xie LH, Hu PS. QTL mapping of grain weight in rice and the validation of the QTL qTGW3. 2. Gene. 2013 Sep 15;527(1):201-6.

Thomson MJ, Tai TH, McClung AM, Lai XH, Hinga ME, Lobos KB, Xu Y, Martinez CP, McCouch SR. Mapping quantitative trait loci for yield, yield components and morphological traits in an advanced backcross population between Oryza rufipogon and the Oryza sativa cultivar Jefferson. Theoretical and applied genetics. 2003 Aug;107(3):479-93.

Wacera HR, Lee HS, Kim KM. Identification of Quantitative Trait Loci Associated with Grain Shape Using Cheongchenong/Nagdong Double Haploid Lines in Rice. Plant Breeding and Biotechnology. 2016 May 31;4(2):188-97.

Wacera HR, Safitri FA, Lee HS, Yun BW, Kim KM. Genetic Mapping of QTLs that control grain characteristics in rice (Oryza sativa L.). Journal of Life Science. 2015;25(8):925-31.

Wang Y, Zang J, Sun Y, Ali J, Xu J, Li Z. Background‐independent quantitative trait loci for drought tolerance identified using advanced backcross introgression lines in rice. Crop Science. 2013 Mar;53(2):430-41.

Xu F, Sun X, Chen Y, Huang Y, Tong C, Bao J. Rapid identification of major QTLs associated with rice grain weight and their utilization. PloS one. 2015 Mar 27;10(3):e0122206.

Yun W, Jinping Z, Yong S, Jauhar A, Jianlong X, Zhikang L. Identification of genetic overlaps for salt and drought tolerance using simple sequence repeat markers on an advanced backcross population in rice. Crop science. 2012 Jul;52(4):1583-92.

Zhang H, Zhu YJ, Zhu AD, Fan YY, Huang TX, Zhang JF, Xie HA, Zhuang JY. Fine-mapping of qTGW2, a quantitative trait locus for grain weight in rice (Oryza sativa L.). PeerJ. 2020 Mar 4;8:e8679.

Zhang HW, Fan YY, Zhu YJ, Chen JY, Yu SB, Zhuang JY. Dissection of the qTGW1. 1 region into two tightly-linked minor QTLs having stable effects for grain weight in rice. BMC genetics. 2016 Dec;17(1):1-0.

Zhu Y, Zhang Z, Chen J, Fan Y, Mou T, Tang S, Zhuang J. Fine mapping of qTGW10-20.8, a QTL having important contribution to grain weight variation in rice. The Crop Journal. 2019 Oct 1;7(5):587-97.

Zhu YJ, Sun ZC, Niu XJ, Ying JZ, Fan YY, Mou TM, Tang SQ, Zhuang JY. Dissection of three quantitative trait loci for grain size on the long arm of chromosome 10 in rice (Oryza sativa L.). PeerJ. 2019 May 16;7:e6966.
