# Supplementary material for: Understanding complex genetic architecture of rice grain weight through QTL-meta analysis and candidate gene identification
Source: Sci Rep. 2022 Aug 16;12:13832. doi: 10.1038/s41598-022-17402-w (PMC9381546; doi:10.1038/s41598-022-17402-w)
Supplement: Supplementary file 5 — Supplementary Information 5. [file 41598_2022_17402_MOESM5_ESM.docx]

Supplementary file 2: Genotypes used for validation and their thousand grain weight (g)

| Low TGW set | | | High TGW set | | |
| --- | --- | --- | --- | --- | --- |
| Sl. No. | Genotype ID | TGW (g) | Sl. No. | Genotype ID | TGW (g) |
| 1 | IG-Selection | 12.10 | 1 | Jai Gundi | 26.15 |
| 2 | Adam chini | 9.50 | 2 | Longku Labat | 27.00 |
| 3 | Shamjira | 12.07 | 3 | Niiaw Hawm | 37.27 |
| 4 | Jeeraphool | 11.04 | 4 | IET-2278 | 26.53 |
| 5 | KH-Sakani | 12.20 | 5 | IGKV R-1 | 30.25 |
| 6 | KH-Sakani-W | 10.73 | 6 | Barhasal | 25.01 |
| 7 | Badshahbhob Sel-1 | 12.25 | 7 | Lunishree | 30.29 |
