# Supplementary material for: Understanding complex genetic architecture of rice grain weight through QTL-meta analysis and candidate gene identification
Source: Sci Rep. 2022 Aug 16;12:13832. doi: 10.1038/s41598-022-17402-w (PMC9381546; doi:10.1038/s41598-022-17402-w)
Supplement: Supplementary file 6 — Supplementary Information 6. [file 41598_2022_17402_MOESM6_ESM.docx]

Details of genes and their positions within the region of MQTL3.1

| Sl No. | Gene ID | Position |
| --- | --- | --- |
| 1 | LOC_Os03g17410 | 9685865 - 9680890 |
| 2 | LOC_Os03g17420 | 9691282 - 9693337 |
| 3 | LOC_Os03g17426 | 9695042 - 9695761 |
| 4 | LOC_Os03g17432 | 9699177 - 9700391 |
| 5 | LOC_Os03g17440 | 9703425 - 9700359 |
| 6 | LOC_Os03g17450 | 9703689 - 9707179 |
| 7 | LOC_Os03g17460 | 9710092 - 9708206 |
| 8 | LOC_Os03g17470 | 9714748 - 9712370 |
| 9 | LOC_Os03g17480 | 9719907 - 9717252 |
| 10 | LOC_Os03g17490 | 9721925 - 9725003 |
| 11 | LOC_Os03g17500 | 9727544 - 9725271 |
| 12 | LOC_Os03g17510 | 9732973 - 9730414 |
| 13 | LOC_Os03g17520 | 9737565 - 9733146 |
| 14 | LOC_Os03g17530 | 9739025 - 9739582 |
| 15 | LOC_Os03g17540 | 9743501 - 9751248 |
| 16 | LOC_Os03g17550 | 9755757 - 9751680 |
| 17 | LOC_Os03g17560 | 9757006 - 9759331 |
| 18 | LOC_Os03g17570 | 9768656 - 9759479 |
| 19 | LOC_Os03g17580 | 9782590 - 9781434 |
| 20 | LOC_Os03g17590 | 9788821 - 9783786 |
| 21 | LOC_Os03g17600 | 9788873 - 9791862 |
| 22 | LOC_Os03g17610 | 9797352 - 9799915 |
| 23 | LOC_Os03g17634 | 9822129 - 9797352 |
| 24 | LOC_Os03g17660 | 9822716 - 9826979 |
| 25 | LOC_Os03g17670 | 9828148 - 9828898 |
| 26 | LOC_Os03g17680 | 9832662 - 9833183 |
| 27 | LOC_Os03g17690 | 9843327 - 9846747 |
| 28 | LOC_Os03g17700 | 9850473 - 9847700 |
| 29 | LOC_Os03g17710 | 9856043 - 9852712 |
| 30 | LOC_Os03g17720 | 9860450 - 9860872 |
| 31 | LOC_Os03g17730 | 9863815 - 9868777 |
| 32 | LOC_Os03g17740 | 9872205 - 9876595 |
| 33 | LOC_Os03g17750 | 9877391 - 9877003 |
| 34 | LOC_Os03g17760 | 9880930 - 9878025 |
| 35 | LOC_Os03g17770 | 9891425 - 9890412 |
| 36 | LOC_Os03g17780 | 9893421 - 9896633 |
| 37 | LOC_Os03g17790 | 9900400 - 9899758 |
| 38 | LOC_Os03g17800 | 9907008 - 9904420 |
| 39 | LOC_Os03g17810 | 9917286 - 9915938 |
| 40 | LOC_Os03g17820 | 9922834 - 9921045 |
| 41 | LOC_Os03g17830 | 9929471 - 9929992 |
| 42 | LOC_Os03g17840 | 9933582 - 9940058 |
| 43 | LOC_Os03g17850 | 9946381 - 9952426 |
| 44 | LOC_Os03g17860 | 9955138 - 9952788 |
| 45 | LOC_Os03g17870 | 9957429 - 9958013 |
| 46 | LOC_Os03g17880 | 9961503 - 9960708 |
| 47 | LOC_Os03g17890 | 9965373 - 9965781 |
| 48 | LOC_Os03g17900 | 9972495 - 9977764 |
| 49 | LOC_Os03g17910 | 9978906 - 9981030 |
| 50 | LOC_Os03g17920 | 9981239 - 9986561 |
| 51 | LOC_Os03g17930 | 9987160 - 9990388 |
| 52 | LOC_Os03g17940 | 9993307 - 9990512 |
| 53 | LOC_Os03g17950 | 9994917 - 9998913 |
| 54 | LOC_Os03g17960 | 9999633 - 10002142 |
| 55 | LOC_Os03g17970 | 10003644 - 10003190 |
| 56 | LOC_Os03g17980 | 10006811 - 10011946 |
| 57 | LOC_Os03g17990 | 10016468 - 10019739 |
| 58 | LOC_Os03g18000 | 10024221 - 10024787 |
| 59 | LOC_Os03g18010 | 10025401 - 10027931 |
| 60 | LOC_Os03g18020 | 10028105 - 10029116 |
| 61 | LOC_Os03g18030 | 10044429 - 10038840 |
| 62 | LOC_Os03g18040 | 10044705 - 10045796 |
| 63 | LOC_Os03g18050 | 10057658 - 10058281 |
| 64 | LOC_Os03g18060 | 10071657 - 10062717 |
| 65 | LOC_Os03g18070 | 10083451 - 10080248 |
| 66 | LOC_Os03g18080 | 10097873 - 10090149 |
| 67 | LOC_Os03g18090 | 10100521 - 10100940 |
| 68 | LOC_Os03g18100 | 10102703 - 10102014 |
| 69 | LOC_Os03g18110 | 10105981 - 10108026 |
| 70 | LOC_Os03g18120 | 10116219 - 10120924 |
| 71 | LOC_Os03g18130 | 10124384 - 10119873 |
| 72 | LOC_Os03g18140 | 10147977 - 10153118 |
| 73 | LOC_Os03g18150 | 10163441 - 10165477 |

Details of genes and their positions within the region of MQTL3.2

| Sl No. | Gene ID | Position |
| --- | --- | --- |
| 1 | LOC_Os03g41510 | 23086275 - 23090306 |
| 2 | LOC_Os03g41530 | 23096386 - 23093139 |
| 3 | LOC_Os03g41540 | 23100472 - 23101690 |
| 4 | LOC_Os03g41570 | 23119044 - 23122456 |
| 5 | LOC_Os03g41580 | 23123593 - 23123059 |
| 6 | LOC_Os03g41600 | 23127983 - 23128906 |
| 7 | LOC_Os03g41612 | 23140230 - 23135473 |
| 8 | LOC_Os03g41624 | 23142781 - 23147227 |
| 9 | LOC_Os03g41640 | 23150566 - 23149433 |
| 10 | LOC_Os03g41650 | 23153673 - 23151315 |
| 11 | LOC_Os03g41662 | 23156099 - 23155593 |
| 12 | LOC_Os03g41675 | 23159127 - 23157870 |
| 13 | LOC_Os03g41690 | 23165458 - 23165841 |
| 14 | LOC_Os03g41700 | 23170084 - 23167124 |
| 15 | LOC_Os03g41710 | 23176315 - 23173062 |
| 16 | LOC_Os03g41720 | 23177506 - 23176661 |
| 17 | LOC_Os03g41740 | 23183608 - 23187458 |
| 18 | LOC_Os03g41750 | 23189236 - 23190465 |
| 19 | LOC_Os03g41770 | 23198427 - 23192525 |
| 20 | LOC_Os03g41780 | 23201176 - 23207379 |
| 21 | LOC_Os03g41790 | 23208034 - 23210281 |
| 22 | LOC_Os03g41800 | 23213785 - 23217778 |
| 23 | LOC_Os03g41810 | 23219212 - 23218781 |
| 24 | LOC_Os03g41820 | 23221927 - 23221526 |
| 25 | LOC_Os03g41830 | 23232645 - 23227125 |
| 26 | LOC_Os03g41840 | 23235754 - 23234414 |
| 27 | LOC_Os03g41850 | 23240408 - 23239272 |
| 28 | LOC_Os03g41860 | 23247136 - 23244333 |
| 29 | LOC_Os03g41870 | 23249588 - 23248780 |
| 30 | LOC_Os03g41890 | 23262074 - 23262832 |
| 31 | LOC_Os03g41879 | 23264876 - 23264430 |
| 32 | LOC_Os03g41910 | 23278859 - 23279095 |
| 33 | LOC_Os03g41920 | 23285567 - 23280702 |
| 34 | LOC_Os03g41932 | 23291191 - 23290579 |
| 35 | LOC_Os03g41940 | 23294912 - 23294200 |
| 36 | LOC_Os03g41960 | 23318841 - 23303545 |
| 37 | LOC_Os03g41970 | 23321492 - 23320512 |
| 38 | LOC_Os03g41980 | 23322635 - 23328795 |
| 39 | LOC_Os03g41990 | 23332413 - 23329181 |
| 40 | LOC_Os03g42000 | 23333676 - 23333353 |
| 41 | LOC_Os03g42010 | 23334978 - 23340901 |
| 42 | LOC_Os03g42020 | 23341805 - 23347331 |
| 43 | LOC_Os03g42030 | 23351131 - 23351813 |
| 44 | LOC_Os03g42040 | 23360680 - 23353232 |
| 45 | LOC_Os03g42050 | 23382556 - 23368991 |
| 46 | LOC_Os03g42060 | 23387693 - 23387051 |
| 47 | LOC_Os03g42070 | 23412753 - 23410207 |
| 48 | LOC_Os03g42080 | 23417451 - 23417831 |
| 49 | LOC_Os03g42090 | 23425245 - 23424832 |
| 50 | LOC_Os03g42100 | 23434712 - 23433316 |
| 51 | LOC_Os03g42110 | 23442337 - 23438157 |
| 52 | LOC_Os03g42120 | 23444572 - 23446715 |
| 53 | LOC_Os03g42130 | 23452171 - 23450164 |
| 54 | LOC_Os03g42140 | 23453889 - 23453396 |
| 55 | LOC_Os03g42150 | 23457864 - 23455130 |
| 56 | LOC_Os03g42170 | 23461605 - 23460943 |
| 57 | LOC_Os03g42180 | 23463301 - 23462933 |
| 58 | LOC_Os03g42190 | 23466453 - 23464716 |
| 59 | LOC_Os03g42200 | 23475827 - 23472278 |
| 60 | LOC_Os03g42210 | 23484619 - 23484996 |
| 61 | LOC_Os03g42220 | 23485491 - 23490741 |
| 62 | LOC_Os03g42230 | 23491995 - 23496324 |
| 63 | LOC_Os03g42235 | 23496334 - 23497401 |
| 64 | LOC_Os03g42240 | 23499435 - 23503720 |
| 65 | LOC_Os03g42259 | 23513579 - 23512612 |
| 66 | LOC_Os03g42270 | 23521799 - 23516352 |
| 67 | LOC_Os03g42280 | 23523560 - 23528315 |
| 68 | LOC_Os03g42284 | 23530602 - 23530061 |
| 69 | LOC_Os03g42290 | 23533463 - 23538865 |
| 70 | LOC_Os03g42310 | 23541015 - 23542945 |
| 71 | LOC_Os03g42320 | 23547668 - 23545317 |
| 72 | LOC_Os03g42334 | 23555365 - 23555901 |
| 73 | LOC_Os03g42350 | 23556180 - 23562967 |
| 74 | LOC_Os03g42360 | 23566454 - 23565984 |
| 75 | LOC_Os03g42370 | 23567544 - 23577244 |
| 76 | LOC_Os03g42380 | 23588045 - 23577861 |
| 77 | LOC_Os03g42400 | 23592134 - 23592585 |
| 78 | LOC_Os03g42410 | 23595409 - 23597429 |
| 79 | LOC_Os03g42420 | 23609771 - 23611885 |
| 80 | LOC_Os03g42430 | 23613955 - 23616375 |
| 81 | LOC_Os03g42440 | 23620260 - 23616738 |
| 82 | LOC_Os03g42450 | 23621303 - 23620827 |
| 83 | LOC_Os03g42464 | 23630649 - 23623464 |
| 84 | LOC_Os03g42480 | 23640082 - 23633268 |
| 85 | LOC_Os03g42490 | 23642211 - 23641888 |
| 86 | LOC_Os03g42500 | 23645268 - 23644298 |
| 87 | LOC_Os03g42510 | 23651394 - 23647892 |
| 88 | LOC_Os03g42520 | 23655935 - 23658301 |
| 89 | LOC_Os03g42530 | 23658542 - 23667612 |
| 90 | LOC_Os03g42540 | 23671609 - 23674781 |
| 91 | LOC_Os03g42550 | 23689494 - 23691731 |
| 92 | LOC_Os03g42569 | 23694358 - 23704806 |
| 93 | LOC_Os03g42590 | 23705874 - 23705188 |

Details of genes and their positions within the region of MQTL3.3

| Sl No. | Gene ID | Position |
| --- | --- | --- |
| 1 | LOC_Os03g51240 | 29313021 - 29315807 |
| 2 | LOC_Os03g51250 | 29320771 - 29315809 |
| 3 | LOC_Os03g51260 | 29323875 - 29326368 |
| 4 | LOC_Os03g51264 | 29326728 - 29326295 |
| 5 | LOC_Os03g51270 | 29336560 - 29328821 |
| 6 | LOC_Os03g51280 | 29342378 - 29345445 |
| 7 | LOC_Os03g51290 | 29348249 - 29351946 |
| 8 | LOC_Os03g51310 | 29353504 - 29359133 |
| 9 | LOC_Os03g51320 | 29363577 - 29360287 |
| 10 | LOC_Os03g51330 | 29370719 - 29373265 |
| 11 | LOC_Os03g51340 | 29376077 - 29378021 |
| 12 | LOC_Os03g51350 | 29386200 - 29388063 |
| 13 | LOC_Os03g51360 | 29390214 - 29393259 |
