# Supplementary material for: Understanding complex genetic architecture of rice grain weight through QTL-meta analysis and candidate gene identification
Source: Sci Rep. 2022 Aug 16;12:13832. doi: 10.1038/s41598-022-17402-w (PMC9381546; doi:10.1038/s41598-022-17402-w)
Supplement: Supplementary file 7 — Supplementary Information 7. [file 41598_2022_17402_MOESM7_ESM.docx]

Supplementary file 4: Functional details of conserved domains of important genes identified under MQTL region

| S. No. | Gene | MQTL No. | RAP Gene ID | Conserved Domain | Gene details | Function |
| --- | --- | --- | --- | --- | --- | --- |
| 1 | LOC_Os03g17410 | MQTL3.1 | Os03g0282232; Os03g0282300 | PX_domain super family |  | phosphoinositide (PI) binding module involved in targeting proteins to membranes |
| 2 | LOC_Os03g17420 | MQTL3.1 |  | transposon protein |  |  |
| 3 | LOC_Os03g17426 | MQTL3.1 | Os03g0282600 |  |  | RNA helicase |
| 4 | LOC_Os03g17432 | MQTL3.1 | Os03g0282700 | HrpA super family | HrpA-like RNA helicase [Translation, ribosomal structure and biogenesis] | pre-mRNA-splicing factor ATP-dependent RNA helicase DHX16; DEAD-like helicase, N-terminal domain containing protein |
| 5 | LOC_Os03g17440 | MQTL3.1 | Os03g0282800 |  |  |  |
| 6 | LOC_Os03g17450 | MQTL3.1 | Os03g0282900 | Ribosomal protein S24e family protein |  | nucleotide binding protein, involved in post-transcriptional gene expression processes including mRNA and rRNA processing, RNA export, and RNA stability |
| 7 | LOC_Os03g17460 | MQTL3.1 | Os03g0283000 | C-terminal, alpha helical domain of Class Lambda Glutathione S-transferases | lambda class glutathione S-transferase 3 | involved in cellular detoxification by catalyzing the conjugation of glutathione (GSH) with a wide range of endogenous and xenobiotic alkylating agents, including carcinogens, therapeutic drugs, environmental toxins, and products of oxidative stress |
| 8 |  |  |  | Glutathione S-transferase, N-terminal domain |  |  |
| 9 | LOC_Os03g17470 | MQTL3.1 | Os03g0283100 |  | SAME AS PREVIOS |  |
| 10 | LOC_Os03g17480 | MQTL3.1 | Os03g0283200 |  | SAME AS PREVIOS |  |
| 11 | LOC_Os03g17490 | MQTL3.1 | Os03g0283300 | Tho1/MOS11 C-terminal domain |  | THO is a multi-protein complex involved in the formation of messenger ribonuclear particles (mRNPs) by coupling transcription with mRNA processing and export. |
| 12 | LOC_Os03g17500 | MQTL3.1 | Os03g0283400 |  |  |  |
| 13 | LOC_Os03g17510 | MQTL3.1 | Os03g0283500 | pentatricopeptide repeat domain |  | PENTATRICOPEPTIDE REPEAT-CONTAINING PROTEIN |
| 14 | LOC_Os03g17520 | MQTL3.1 | Os03g0283600 | CDP-alcohol phosphatidyltransferase |  | CDP-alcohol phosphatidyltransferase, ability to catalyze the displacement of CMP from a CDP-alcohol by a second alcohol with formation of a phosphodiester bond and concomitant breaking of a phosphoride anhydride bond |
| 15 | LOC_Os03g17540 | MQTL3.1 | Os03g0283800 | TBC domain containing protein |  | performs a GTP-activator activity on Rab-like GTPases. |
| 16 |  |  |  | COG4372 super family |  |  |
| 17 | LOC_Os03g17550 | MQTL3.1 | Os03g0283900 | Catalytic domain of the Serine/Threonine kinases, Interleukin-1 Receptor Associated Kinases | serine/threonine-protein kinase; Serine/threonine-protein kinase PBS1 | plant receptor-like kinases (RLKs) including Arabidopsis thaliana BAK1 and CLAVATA1 (CLV1). BAK1 functions in BR (brassinosteroid)-regulated plant development and in pathways involved in plant resistance to pathogen infection and herbivore attack. CLV1, directly binds small signaling peptides, CLAVATA3 (CLV3) and CLAVATA3/EMBRYO SURROUNDING REGI0N (CLE), to restrict stem cell proliferation: the CLV3-CLV1-WUS (WUSCHEL) module influences stem cell maintenance in the shoot apical meristem, and the CLE40 (CLAVATA3/EMBRYO SURROUNDING REGION40) -ACR4 (CRINKLY4) -CLV1- WOX5 (WUSCHEL-RELATED HOMEOBOX5) module at the root apical meristem. The IRAK subfamily is part of a larger superfamily that includes the catalytic domains of other protein STKs, protein tyrosine kinases, RIO kinases, aminoglycoside phosphotransferase, choline kinase, and phosphoinositide 3-kinase. |
| 18 | LOC_Os03g17560 | MQTL3.1 | Os03g0284000 | P-loop_NTPase super family | conserved nucleotide phosphate-binding motif, also referred to as the Walker A motif (GxxxxGK[S/T], where x is any residue), and the Walker B motif (hhhh[D/E], where h is a hydrophobic residue). The Walker A and B motifs bind the beta-gamma phosphate moiety of the bound nucleotide (typically ATP or GTP) and the Mg2+ cation, respectively. | The P-loop NTPases are involved in diverse cellular functions, and they can be divided into two major structural classes: the KG (kinase-GTPase) class which includes Ras-like GTPases and its circularly permutated YlqF-like; and the ASCE (additional strand catalytic E) class which includes ATPase Binding Cassette (ABC), DExD/H-like helicases, 4Fe-4S iron sulfur cluster binding proteins of NifH family, RecA-like F1-ATPases, and ATPases Associated with a wide variety of Activities (AAA). Also included are a diverse set of nucleotide/nucleoside kinase families. |
| 19 | LOC_Os03g17570 | MQTL3.1 | Os03g0284100 | psREC_PRR |  | response regulator receiver domain containing protein, controls the pace of the central oscillator of the circadian clock, an endogenous time-keeping mechanism that enables organisms to adapt to external daily cycles |
| 20 |  |  |  | CCT motif; |  | contains a putative nuclear localization signal |
| 21 | LOC_Os03g17580 | MQTL3.1 | Os03g0284400 | Ribosomal protein L10 family | Ribosomal protein L10-like | Similar to Ribosomal protein L10-like, composed of bacterial 50S ribosomal protein and eukaryotic mitochondrial 39S ribosomal protein, L10 |
| 22 | LOC_Os03g17590 | MQTL3.1 | Os03g0284500 | HAD-superfamily subfamily IIA hydrolase, CECR5 super family |  | HAD-superfamily subfamily IIA hydrolase, CECR5 protein, haloacid dehalogenase superfamily of aspartate-nucleophile hydrolases, |
| 23 | LOC_Os03g17600 | MQTL3.1 | Os03g0284600 | Thioredoxin-like fold domain containing protein, | Members of the superfamily that do not function as PDOs but contain a TRX-fold domain include phosducins, peroxiredoxins, glutathione (GSH) peroxidases, SCO proteins, GSH transferases (GST, N-terminal domain), arsenic reductases, TRX-like ferredoxins and calsequestrin, among others. | Thioredoxin-like fold domain containing protein, function as protein disulfide oxidoreductases (PDOs), altering the redox state of target proteins via the reversible oxidation of their active site dithiol, |
| 24 | LOC_Os03g17610 | MQTL3.1 | Os03g0284800 | DNA topoisomerase IV subunit A; | Spo11/DNA topoisomerase VI, subunit A family protein | TOPOISOMERASE 6 SUBUNIT A3 |
| 25 | LOC_Os03g17634 | MQTL3.1 | Os03g0284900 | Annexin repeat, conserved site domain containing protein | Reverse transcriptase (RNA-dependent DNA polymerase); gag-polypeptide of LTR copia-type; PPR repeat family, GAG-pre-integrase domain, Ty3/Gypsy family of RNase HI in long-term repeat retroelements, |  |
| 26 | LOC_Os03g17690 | MQTL3.1 | Os03g0285700 | plant_peroxidase_like super family; Cytosolic ascorbate peroxidase, Salt tolerance; ASCORBATE PEROXIDASE 1 | Heme-dependent peroxidases similar to plant peroxidases; catalyzes a multistep oxidative reaction involving hydrogen peroxide as the electron acceptor |  |
| 27 | LOC_Os03g17700 | MQTL3.1 | Os03g0285800 | PKc_like super family | Mitogen-activated protein kinase 5, BTH-induced MAPK 1; The protein kinase superfamily is mainly composed of the catalytic domains of serine/threonine-specific and tyrosine-specific protein kinases | Disease resistance, Abiotic stress tolerance, Positively regulation of drought, salt and cold tolerance, These proteins catalyze the transfer of the gamma-phosphoryl group from ATP to hydroxyl groups in specific substrates such as serine, threonine, or tyrosine residues of proteins |
| 28 | LOC_Os03g17710 | MQTL3.1 | Os03g0285900 | universal minicircle sequence binding protein | Similar to Splicing factor RSZ33 | RS domain with zinc knuckle protein 37b, RSZ subfamily protein 37b, RS2Z subfamily protein 38 |
| 29 |  |  |  | RNA recognition motif (RRM) superfamily |  |  |
| 30 | LOC_Os03g17720 | MQTL3.1 | Os03g0286100 | RING_Ubox super family | RING finger is a specialized type of Zn-finger of 40 to 60 residues that binds two atoms of zinc. | RING finger/U-box-containing proteins are a group of diverse proteins with a variety of cellular functions, including oncogenesis, development, viral replication, signal transduction, the cell cycle and apoptosis. |
| 31 | LOC_Os03g17730 | MQTL3.1 | Os03g0286200 | arogenate dehydratase | Similar to Prephenate dehydratase-like |  |
| 32 | LOC_Os03g17740 | MQTL3.1 | Os03g0286300 | Triose-phosphate Transporter family | Similar to Phosphate/phosphoenolpyruvate translocator protein-like | includes transporters with a specificity for triose phosphate. |
| 33 | LOC_Os03g17750 | MQTL3.1 |  | Cellulose synthase-like protein |  |  |
| 34 | LOC_Os03g17760 | MQTL3.1 | Os03g0286500 | RNA recognition motif (RRM) superfamily | Similar to RNA-binding region containing protein 1 | involved in post-transcriptional gene expression processes including mRNA and rRNA processing, RNA export, and RNA stability |
| 35 | LOC_Os03g17770 | MQTL3.1 | Os03g0286700 | Major Facilitator Superfamily |  | The Major Facilitator Superfamily (MFS) is a large and diverse group of secondary transporters that includes uniporters, symporters, and antiporters. MFS proteins facilitate the transport across cytoplasmic or internal membranes of a variety of substrates including ions, sugar phosphates, drugs, neurotransmitters, nucleosides, amino acids, and peptides. |
| 36 | LOC_Os03g17780 | MQTL3.1 | Os03g0286800 | WD40 domain | Similar to WD and tetratricopeptide repeats protein 1. | functions including adaptor/regulatory modules in signal transduction, pre-mRNA processing and cytoskeleton assembly; |
| 37 | LOC_Os03g17790 | MQTL3.1 | Os03g0286900 |  | RCI2 (rare cold-inducible 2) family protein |  |
| 38 | LOC_Os03g17800 | MQTL3.1 | Os03g0287100 | START/RHO_alpha_C/PITP/Bet_v1/CoxG/CalC (SRPBCC) ligand-binding domain superfamily | Similar to Phosphatidylinositol transfer protein |  |
| 39 | LOC_Os03g17810 | MQTL3.1 | Os03g0287400 | Lateral organ boundaries (LOB) domain | Similar to LOB domain protein 4 | The lateral organ boundaries (LOB) gene encodes a plant-specific protein of unknown function that is expressed at the adaxial base of initiating lateral organs |
| 40 | LOC_Os03g17820 | MQTL3.1 |  |  | 4-hydroxyphenylacetate decarboxylase activase | 4-hydroxyphenylacetate decarboxylase activase is a radical SAM enzyme, found in anaerobic bacteria where 4-hydroxyphenylacetate decarboxylase occurs and required to prepare the glycyl radical active site of the enzyme. |
| 41 | LOC_Os03g17830 | MQTL3.1 |  |  | etrotransposon protein, putative, Ty3-gypsy subclass |  |
| 42 | LOC_Os03g17840 | MQTL3.1 | Os03g0287600; Os03g0287700 | Domain of unknown function |  |  |
| 43 | LOC_Os03g17850 | MQTL3.1 | Os03g0287800 | Glyco_tranf_GTA_type super family | Similar to beta3-glucuronyltransferase | Glycosyltransferases (GTs) are enzymes that synthesize oligosaccharides, polysaccharides, and glycoconjugates by transferring the sugar moiety from an activated nucleotide-sugar donor to an acceptor molecule, which may be a growing oligosaccharide, a lipid, or a protein. |
| 44 | LOC_Os03g17860 | MQTL3.1 | Os03g0287900 | Thioredoxin_like super family | Similar to Protein disulfide isomerase | function as protein disulfide oxidoreductases (PDOs) |
| 45 | LOC_Os03g17870 | MQTL3.1 | Os03g0288000 | Metallothionein | METALLOTHIONEIN I-1B | Type 1 metallothionein isoform, Positive regulation of resistance to blast disease; bind to heavy metals |
| 46 | LOC_Os03g17880 | MQTL3.1 | Os03g0288100 |  |  |  |
| 47 | LOC_Os03g17890 | MQTL3.1 |  |  |  |  |
| 48 | LOC_Os03g17900 | MQTL3.1 | Os03g0288300 | Abhydrolase super family | Alpha/beta hydrolase fold-1 domain containing protein | A functionally diverse superfamily containing proteases, lipases, peroxidases, esterases, epoxide hydrolases and dehalogenases. |
| 49 | LOC_Os03g17910 | MQTL3.1 | Os03g0288400 | Cytochrome c oxidase assembly protein COX16 | Similar to kinesin like protein | involved in assembly of cytochrome oxidase |
| 50 | LOC_Os03g17920 | MQTL3.1 | Os03g0288500 | Pseudouridine synthase domain |  | Pseudouridine synthases catalyze the isomerization of specific uridines in an RNA molecule to pseudouridines |
| 51 | LOC_Os03g17930 | MQTL3.1 | Os03g0288600 | Taxilin super family | Muscle derived-like protein. (Os03t0288600-01);Similar to SKIP interacting protein 6 | Myosin-like coiled-coil protein; |
| 52 | LOC_Os03g17940 | MQTL3.1 | Os03g0288700 | PAP2_dolichyldiphosphatase | Phosphatidic acid phosphatase type 2/haloperoxidase domain containing protein | Dolichyldiphosphatase is a membrane-associated protein located in the endoplasmic reticulum and hydrolyzes dolichyl pyrophosphate, The enzyme is necessary for maintaining proper levels of dolichol-linked oligosaccharides and protein N-glycosylation, and might play a role in re-utilization of the glycosyl carrier lipid for additional rounds of lipid intermediate biosynthesis after its release during protein N-glycosylation reactions |
| 53 | LOC_Os03g17950 | MQTL3.1 | Os03g0288800 | Cytochrome B561, N terminal | Cytochrome B561-related domain containing protein | found in the N terminal region of cytochrome B561 |
| 54 | LOC_Os03g17980 | MQTL3.1 | Os03g0289100 | PKc_like super family | Serine/threonine protein kinase | composed of the catalytic domains of serine/threonine-specific and tyrosine-specific protein kinases. |
| 55 |  |  |  | UBA_SnRK1_plant | Serine/threonine protein kinase | found in the plant sucrose nonfermenting-1-related kinase (SnRK1) proteins |
| 56 |  |  |  | AMPKA_C_like super family | Serine/threonine protein kinase | composed of AMPKs, microtubule-associated protein/microtubule affinity regulating kinases (MARKs), |
| 57 | LOC_Os03g17990 | MQTL3.1 | Os03g0289200 | Yip1 domain | Similar to H/ACA ribonucleoprotein complex subunit 1-like protein | integral membrane domain contains four transmembrane alpha helices. The domain is characterized by the motifs DLYGP and GY. The Yip1 protein is a golgi protein involved in vesicular transport that interacts with GTPases. |
| 58 | LOC_Os03g18000 | MQTL3.1 | Os03g0289300 | phosphoinositide phospholipase C 4; phosphoinositide phospholipase C | Phosphoinositide-specific phospholipase C |  |
| 59 | LOC_Os03g18010 | MQTL3.1 | Os03g0289300 | same as previous |  |  |
| 60 | LOC_Os03g18020 | MQTL3.1 | Os03g0289400 |  | Rhodanese-like domain containing protein |  |
| 61 | LOC_Os03g18030 | MQTL3.1 | Os03g0289800 | leucoanthocyanidin dioxygenase like protein | Similar to Leucoanthocyanidin dioxygenase-like protein |  |
| 62 | LOC_Os03g18050 | MQTL3.1 | Os03g0290100 | Auxin_inducible |  | This family consists of the protein products of the ARG7 auxin responsive genes family |
| 63 |  |  |  | Pectinesterase super family |  | Pectinesterase |
| 64 | LOC_Os03g18060 | MQTL3.1 |  | PMD super family | in a variety of transposases. |  |
| 65 |  |  |  | DBD_Tnp_Mut super family | presumed to be the transposases for Mutator transposable elements |  |
| 66 |  |  |  | DDE_Tnp_ISL3 super family | transposase proteins are necessary for efficient DNA transposition. |  |
| 67 | LOC_Os03g18070 | MQTL3.1 | Os03g0290300 | omega-3 fatty acid desaturase | Similar to W-3 fatty acid desaturase |  |
| 68 | LOC_Os03g18080 | MQTL3.1 | Os03g0290500 | SacI homology domain | Synaptojanin, N-terminal domain containing protein |  |
| 69 |  |  |  | Phosphoinositide polyphosphatase |  | Signal transduction mechanisms |
| 70 | LOC_Os03g18110 | MQTL3.1 | Os03g0290900 | PLN02629 super family | Protein of unknown function DUF231, plant domain containing protein | powdery mildew resistance 5 |
| 71 | LOC_Os03g18120 | MQTL3.1 | Os03g0291200 | SGNH_hydrolase super family | Protein of unknown function DUF231, plant domain containing protein | SGNH_hydrolase, or GDSL_hydrolase, is a diverse family of lipases and esterases |
| 72 |  |  |  | PMR5N |  | The plant family with PMR5, ESK1, TBL3 etc have a N-terminal C rich predicted sugar binding domain followed by the PC-Esterase (acyl esterase) domain |
| 73 | LOC_Os03g18130 | MQTL3.1 | Os03g0291500 | asparagine synthetase-like protein | Asparagine synthetase, Biosynthesis of asparagine following the supply of ammonium |  |
| 74 |  |  |  | asparagine synthase (glutamine-hydrolyzing) |  |  |
| 75 | LOC_Os03g18140 | MQTL3.1 | Os03g0291800 | SGNH_hydrolase super family |  | SGNH_hydrolase, or GDSL_hydrolase, is a diverse family of lipases and esterases. |
| 76 |  |  |  | PMR5N |  | the plant family with PMR5, ESK1, TBL3 etc have a N-terminal C rich predicted sugar binding domain followed by the PC-Esterase (acyl esterase) domain |
| 77 | LOC_Os03g18150 | MQTL3.1 | Os03g0292100 | Serine/threonine phosphatases, family 2C, catalytic domain |  | Serine/threonine phosphatases |
| 78 | LOC_Os03g41510 | MQTL3.2 | Os03g0611200 | Aldo-keto reductase (AKR) superfamily | Aldo/keto reductase family protein | Aldo-keto reductases (AKRs) are a superfamily of soluble NAD(P)(H) oxidoreductases whose chief purpose is to reduce aldehydes and ketones to primary and secondary alcohols. |
| 79 | LOC_Os03g41570 | MQTL3.2 |  | Transposase | transposon gene | Transposase proteins are necessary for efficient DNA transposition |
| 80 |  |  |  | FHY3 super family |  | Protein FAR-RED ELONGATED HYPOCOTYL 3; Provisional |
| 81 |  |  |  | DBD_Tnp_Mut super family |  | This region is found in plant proteins that are presumed to be the transposases for Mutator transposable elements |
| 82 |  |  |  | plant mutator transposase zinc finger |  | plant mutator transposase zinc finger |
| 83 | LOC_Os03g41580 | MQTL3.2 |  | zf-GRF super family | transposon protein, putative, CACTA, En/Spm sub-class; GRF zinc finger | This presumed zinc binding domain is found in a variety of DNA-binding proteins. It seems likely that this domain is involved in nucleic acid binding. |
| 84 | LOC_Os03g41600 | MQTL3.2 | Os03g0612400 | Lateral organ boundaries (LOB) domain | Similar to ASL1 | The lateral organ boundaries (LOB) gene encodes a plant-specific protein of unknown function that is expressed at the adaxial base of initiating lateral organs. |
| 85 |  |  |  | Wound-induced protein |  | The proteins in the family are often annotated as wound-induced proteins |
| 86 | LOC_Os03g41612 | MQTL3.2 | Os03g0612600 | Ribosomal protein TL5, C-terminal domain; | Similar to Ribosomal 5S rRNA E-loop binding protein Ctc/L25/TL5 containing protein | contains the C-terminal domain of ribosomal protein TL5 |
| 87 |  |  |  | 50S ribosomal protein L25/general stress protein Ctc |  |  |
| 88 |  |  |  | Ribosomal L25/TL5/CTC N-terminal 5S rRNA binding domain |  | L25 is a single-domain protein, homologous to the N-terminal domain of TL5 and CTC |
| 89 | LOC_Os03g41662 | MQTL3.2 |  |  |  |  |
| 90 | LOC_Os03g41675 | MQTL3.2 | Os03g0613100 | Superfamily of activating enzymes (E1) of the ubiquitin-like proteins |  | Superfamily of activating enzymes (E1) of the ubiquitin-like proteins. This family includes classical ubiquitin-activating enzymes E1, ubiquitin-like (ubl) activating enzymes and other mechanistic homologes, like MoeB, Thif1 and others |
| 91 | LOC_Os03g41800 | MQTL3.2 | Os03g0614250 |  | Protein FAR-RED ELONGATED HYPOCOTYL 3 | Protein FAR-RED ELONGATED HYPOCOTYL 3 |
| 92 | LOC_Os03g41920 | MQTL3.2 | Os03g0615300 | Protein of unknown function (DUF1618) | Protein of unknown function DUF1618 domain containing protein |  |
| 93 | LOC_Os03g42010 | MQTL3.2 | Os03g0616300 | DNA polymerase IV; | Similar to DNA polymerase kappa | DNA Polymerase |
| 94 | LOC_Os03g42020 | MQTL3.2 | Os03g0616400 | HAD_like super family | Haloacid Dehalogenase-like Hydrolases |  |
| 95 |  |  |  | ATPase-IIB_Ca super family | plasma-membrane calcium-translocating P-type ATPase | This model describes the P-type ATPase responsible for translocating calcium ions across the plasma membrane of eukaryotes, out of the cell |
| 96 | LOC_Os03g42030 | MQTL3.2 | Os03g0616450 |  |  |  |
| 97 | LOC_Os03g42040 | MQTL3.2 | Os03g0616500 | Condensin II non structural maintenance of chromosomes subunit; | Similar to HEAT repeat family protein | This family is part of a non-SMC subunit of condensin II which is involved in maintenance of the structural integrity of chromosomes |
| 98 | LOC_Os03g42050 | MQTL3.2 | Os03g0616700 |  | Similar to Importin-beta N-terminal domain containing protein, |  |
| 99 | LOC_Os03g42060 | MQTL3.2 |  |  |  |  |
| 100 | LOC_Os03g42070 | MQTL3.2 | Os03g0617500 | Cyclin box fold superfamily | Cyclin, C-terminal domain containing protein | functions in cell-cycle and transcriptional control |
| 101 | LOC_Os03g42110 | MQTL3.2 | Os03g0617900 | Probable N-acetyl-gamma-glutamyl-phosphate reductase | Semialdehyde dehydrogenase, dimerisation region domain containing protein |  |
| 102 | LOC_Os03g42120 | MQTL3.2 | Os03g0618000 |  |  |  |
| 103 | LOC_Os03g42130 | MQTL3.2 | Os03g0618300 | oxidoreductase, 2OG-Fe(II) oxygenase family protein | Isopenicillin N synthase family protein |  |
| 104 | LOC_Os03g42190 | MQTL3.2 | Os03g0618800 | DNA polymerase III subunit gamma/tau |  |  |
| 105 |  |  | Os03g0619002 | PGG super family |  | PGG domain is named for the highly conserved sequence motif found at the startt of the domain |
| 106 |  |  | Os03g0619151 | Dof domain, zinc finger | Similar to Dof domain, zinc finger family protein | Dof domain is a zinc finger DNA-binding domain, that shows resemblance to the Cys2 zinc finger. |
| 107 | LOC_Os03g42220 | MQTL3.2 | Os03g0619400 | chaperonin_like superfamily | Chaperone, tailless complex polypeptide 1 domain containing protein | nvolved in productive folding of proteins |
| 108 |  |  |  | T-complex protein 1 subunit beta |  |  |
| 109 | LOC_Os03g42230 | MQTL3.2 | Os03g0619600 | Plant-specific B3-DNA binding domain | Transcriptional factor B3 family protein | includes the well-characterized auxin response factor (ARF) and the LAV (Leafy cotyledon2 [LEC2]-Abscisic acid insensitive3 [ABI3]-VAL) families, as well as the RAV (Related to ABI3 and VP1) and REM (REproductive Meristem) families. LEC2 and ABI3 have been shown to be involved in seed development, while other members of the LAV family seem to have a more general role, being expressed in many organs during plant development. |
| 110 | LOC_Os03g42235 | MQTL3.2 | Os03g0619700 |  |  |  |
| 111 | LOC_Os03g42240 | MQTL3.2 | Os03g0619800 | Plant-specific B3-DNA binding domain | Transcriptional factor B3 domain containing protein | includes the well-characterized auxin response factor (ARF) and the LAV (Leafy cotyledon2 [LEC2]-Abscisic acid insensitive3 [ABI3]-VAL) families, as well as the RAV (Related to ABI3 and VP1) and REM (REproductive Meristem) families. LEC2 and ABI3 have been shown to be involved in seed development, while other members of the LAV family seem to have a more general role, being expressed in many organs during plant development. |
| 112 | LOC_Os03g42280 | MQTL3.2 | Os03g0620400 | Plant-specific B3-DNA binding domain; | Transcriptional factor B3 domain containing protein | includes the well-characterized auxin response factor (ARF) and the LAV (Leafy cotyledon2 [LEC2]-Abscisic acid insensitive3 [ABI3]-VAL) families, as well as the RAV (Related to ABI3 and VP1) and REM (REproductive Meristem) families. LEC2 and ABI3 have been shown to be involved in seed development, while other members of the LAV family seem to have a more general role, being expressed in many organs during plant development. |
| 113 | LOC_Os03g42290 | MQTL3.2 | Os03g0620500 | Plant-specific B3-DNA binding domain; | Transcriptional factor B3 family protein | includes the well-characterized auxin response factor (ARF) and the LAV (Leafy cotyledon2 [LEC2]-Abscisic acid insensitive3 [ABI3]-VAL) families, as well as the RAV (Related to ABI3 and VP1) and REM (REproductive Meristem) families. LEC2 and ABI3 have been shown to be involved in seed development, while other members of the LAV family seem to have a more general role, being expressed in many organs during plant development. |
| 114 | LOC_Os03g42320 | MQTL3.2 | Os03g0620800 | Sec1 family; | Similar to SEC1-family transport protein SLY1; |  |
| 115 | LOC_Os03g42350 | MQTL3.2 | Os03g0621400 | Ankyrin repeats (3 copies) | TETRATRICOPEPTIDE REPEAT DOMAIN CONTAINING PROTEIN 90 |  |
| 116 |  |  |  | Ankyrin repeats (many copies); |  |  |
| 117 | LOC_Os03g42370 | MQTL3.2 | Os03g0621600 | Plant-specific B3-DNA binding domain; | Transcriptional factor B3 domain containing protein | includes the well-characterized auxin response factor (ARF) and the LAV (Leafy cotyledon2 [LEC2]-Abscisic acid insensitive3 [ABI3]-VAL) families, as well as the RAV (Related to ABI3 and VP1) and REM (REproductive Meristem) families. LEC2 and ABI3 have been shown to be involved in seed development, while other members of the LAV family seem to have a more general role, being expressed in many organs during plant development. |
| 118 |  |  | Os03g0621650 | Plant-specific B3-DNA binding domain; | Transcriptional factor B3 domain containing protein | includes the well-characterized auxin response factor (ARF) and the LAV (Leafy cotyledon2 [LEC2]-Abscisic acid insensitive3 [ABI3]-VAL) families, as well as the RAV (Related to ABI3 and VP1) and REM (REproductive Meristem) families. LEC2 and ABI3 have been shown to be involved in seed development, while other members of the LAV family seem to have a more general role, being expressed in many organs during plant development. |
| 119 | LOC_Os03g42380 | MQTL3.2 | Os03g0621700 | Helitron helicase-like domain at N-terminus | ecognized eukaryotic transposons that are predicted to amplify by a rolling-circle mechanism |  |
| 120 |  |  |  | N-terminal helicase domain of the DEAD-box helicase superfamily | The DEAD-like helicase superfamily is a diverse family of proteins involved in ATP-dependent RNA or DNA unwinding. |  |
| 121 |  |  |  | ATP-dependent exoDNAse (exonuclease V), alpha subunit, helicase superfamily I | ATP-dependent exoDNAse (exonuclease V), alpha subunit, helicase superfamily I [Replication, recombination and repair] |  |
| 122 | LOC_Os03g42400 | MQTL3.2 | Os03g0621900 | Plant-specific B3-DNA binding domain | Transcriptional factor B3 domain containing protein | includes the well-characterized auxin response factor (ARF) and the LAV (Leafy cotyledon2 [LEC2]-Abscisic acid insensitive3 [ABI3]-VAL) families, as well as the RAV (Related to ABI3 and VP1) and REM (REproductive Meristem) families. LEC2 and ABI3 have been shown to be involved in seed development, while other members of the LAV family seem to have a more general role, being expressed in many organs during plant development. |
| 123 | LOC_Os03g42410 | MQTL3.2 | Os03g0621900 | Plant-specific B3-DNA binding domain | Transcriptional factor B3 domain containing protein | includes the well-characterized auxin response factor (ARF) and the LAV (Leafy cotyledon2 [LEC2]-Abscisic acid insensitive3 [ABI3]-VAL) families, as well as the RAV (Related to ABI3 and VP1) and REM (REproductive Meristem) families. LEC2 and ABI3 have been shown to be involved in seed development, while other members of the LAV family seem to have a more general role, being expressed in many organs during plant development. |
| 124 | LOC_Os03g42420 | MQTL3.2 | Os03g0622100 | Plant-specific B3-DNA binding domain; | Transcriptional factor B3 family protein | includes the well-characterized auxin response factor (ARF) and the LAV (Leafy cotyledon2 [LEC2]-Abscisic acid insensitive3 [ABI3]-VAL) families, as well as the RAV (Related to ABI3 and VP1) and REM (REproductive Meristem) families. LEC2 and ABI3 have been shown to be involved in seed development, while other members of the LAV family seem to have a more general role, being expressed in many organs during plant development. |
| 125 | LOC_Os03g42430 | MQTL3.2 | Os03g0622200 | Plant-specific B3-DNA binding domain; | Transcriptional factor B3 family protein | includes the well-characterized auxin response factor (ARF) and the LAV (Leafy cotyledon2 [LEC2]-Abscisic acid insensitive3 [ABI3]-VAL) families, as well as the RAV (Related to ABI3 and VP1) and REM (REproductive Meristem) families. LEC2 and ABI3 have been shown to be involved in seed development, while other members of the LAV family seem to have a more general role, being expressed in many organs during plant development. |
| 126 | LOC_Os03g42440 | MQTL3.2 | Os03g0622300 | Plant-specific B3-DNA binding domain; | Transcriptional factor B3 family protein | includes the well-characterized auxin response factor (ARF) and the LAV (Leafy cotyledon2 [LEC2]-Abscisic acid insensitive3 [ABI3]-VAL) families, as well as the RAV (Related to ABI3 and VP1) and REM (REproductive Meristem) families. LEC2 and ABI3 have been shown to be involved in seed development, while other members of the LAV family seem to have a more general role, being expressed in many organs during plant development. |
| 127 | LOC_Os03g42450 | MQTL3.2 | Os03g0622400 |  | Protein of unknown function (DUF3123) |  |
| 128 | LOC_Os03g42480 | MQTL3.2 | Os03g0622600 | K+-dependent Na+/Ca+ exchanger |  | Transport and binding proteins, Cations and iron carrying compounds |
| 129 |  |  |  | Triose-phosphate Transporter family |  | includes transporters with a specificity for triose phosphate |
| 130 | LOC_Os03g42490 | MQTL3.2 | Os03g0622800 |  | Protein of unknown function (DUF3123) |  |
| 131 | LOC_Os03g42500 | MQTL3.2 | Os03g0622900 |  | Protein of unknown function (DUF3123) |  |
| 132 | LOC_Os03g42510 | MQTL3.2 | Os03g0623000 | GT1, myb-like, SANT family | GT-1, a myb-like protein, is one of the GT trihelix transcription factors. GT-1 binds the GT cis-element of rbcS-3A, a light-induced gene, as a dimer. | GT-1 may respond to light signals via calcium-dependent phosphorylation to create a light-modulated molecular switch. |
| 133 | LOC_Os03g42569 | MQTL3.2 | Os03g0624000 | SANT super family | Similar to KANADI 3 | SWI3, ADA2, N-CoR and TFIIIB' DNA-binding domains. Tandem copies of the domain bind telomeric DNA tandem repeatsas part of the capping complex. Binding is sequence dependent for repeats which contain the G/C rich motif [C2-3 A (CA)1-6]. The domain is also found in regulatory transcriptional repressor complexes where it also binds DNA. |
| 134 | LOC_Os03g51240 | MQTL3.3 | Os03g0722500 | Glyco_hydro super family | Glycoside hydrolase, family 17 protein | Glycosyl hydrolases |
| 135 | LOC_Os03g51250 | MQTL3.3 | Os03g0722600 | Adenylate cyclase-associated CAP domain containing protein | Adenylate cyclase associated (CAP) |  |
| 136 | LOC_Os03g51260 | MQTL3.3 | Os03g0722700 | PLN03142 super family | Probable chromatin-remodeling complex ATPase chain |  |
| 137 |  |  |  | DEAD-like_helicase_N super family | N-terminal helicase domain of the DEAD-box helicase superfamily | The DEAD-like helicase superfamily is a diverse family of proteins involved in ATP-dependent RNA or DNA unwinding. |
| 138 | LOC_Os03g51264 | MQTL3.3 |  |  |  |  |
| 139 | LOC_Os03g51270 | MQTL3.3 | Os03g0722800 |  | Similar to F-box domain containing protein |  |
| 140 | LOC_Os03g51330 | MQTL3.3 | Os03g0723000 | GRAS domain family | Transcription factor, GRAS protein, Regulation of grain shape | Proteins in the GRAS (GAI, RGA, SCR) family are known as major players in gibberellin (GA) signaling, which regulates various aspects of plant growth and development. Mutation of the SCARECROW (SCR) gene results in a radial pattern defect, loss of a ground tissue layer, in the root. The PAT1 protein is involved in phytochrome A signal transduction |
| 141 | LOC_Os03g51350 | MQTL3.3 | Os03g0723400 |  | endosperm-specific gene 53; Similar to UFG2 |  |
