# Supplementary material for: Understanding complex genetic architecture of rice grain weight through QTL-meta analysis and candidate gene identification
Source: Sci Rep. 2022 Aug 16;12:13832. doi: 10.1038/s41598-022-17402-w (PMC9381546; doi:10.1038/s41598-022-17402-w)
Supplement: Supplementary file 8 — Supplementary Information 8. [file 41598_2022_17402_MOESM8_ESM.docx]

Supplementary File 5: Orthologues found in important cereal crops of Poaceae

|  |  |  | Rice | sorghum | barley | Maize | wheat |
| --- | --- | --- | --- | --- | --- | --- | --- |
| MQTL3.1 | LOC_Os03g17460 | IN2-1 protein | Yes | YES | Yes | Yes | Yes |
| MQTL3.1 | LOC_Os03g17470 | IN2-1 protein | Yes | YES | Yes | Yes | Yes |
| MQTL3.1 | LOC_Os03g17480 | IN2-1 protein | Yes | Yes | YES | Yes |  |
| MQTL3.1 | LOC_Os03g17570 | PRR73: Similar to Two-component response regulator-like | Yes | Yes | Yes | Yes | YES |
| MQTL3.1 | LOC_Os03g17610 | OsTOP6A3 - Topoisomerase 6 subunit A homolog 3 | Yes | Yes | Yes | Yes | YES |
| MQTL3.1 | LOC_Os03g17690* | OsAPx1 - Cytosolic Ascorbate Peroxidase encoding gene 1-8 | Yes | Yes | Yes | Yes | YES |
| MQTL3.1 | LOC_Os03g17700 | CGMC_MAPKCGMC_2_ERK.2 - CGMC includes CDA, MAPK, GSK3, and CLKC kinases | Yes | Yes | Yes | Yes | YES |
| MQTL3.1 | LOC_Os03g17780 | WD domain, G-beta repeat domain containing protein | Yes | Yes | Yes | Yes | YES |
| MQTL3.1 | LOC_Os03g17790 | OsRCI2-5 - Putative low temperature and salt responsive protein | Yes | Yes | Yes | Yes |  |
| MQTL3.1 | LOC_Os03g17860* | OsPDIL5-1 protein disulfide isomerase PDIL5-1 | Yes | Yes | Yes | Yes | Yes |
| MQTL3.1 | LOC_Os03g17870 | metallothionein, putative | Yes | Yes | Yes | Yes | Yes |
| MQTL3.1 | LOC_Os03g17980 | CAMK_KIN1/SNF1/Nim1_like_AMPKh.2 - CAMK includes calcium/calmodulin depedent protein kinases | Yes | Yes | Yes | Yes | Yes |
| MQTL3.1 | LOC_Os03g18050* | OsSAUR13 - Auxin-responsive SAUR gene family member | Yes | Yes | Yes | Yes | Yes |
| MQTL3.1 | LOC_Os03g18110 | proteins of unknown function domain containing protein | Yes | Yes | Yes | Yes | Yes |
| MQTL3.1 | LOC_Os03g18120 | proteins of unknown function domain containing protein | Yes | Yes |  | Yes |  |
| MQTL3.1 | LOC_Os03g18130* | asparagine synthetase, putative | Yes | Yes | Yes | Yes | Yes |
| MQTL3.1 | LOC_Os03g18140 | XYLAN O-ACETYLTRANSFERASE 6 | Yes | Yes | Yes | Yes |  |
| MQTL3.1 | LOC_Os03g18150 | protein phosphatase 2C, putative | Yes | Yes | Yes | Yes | Yes |
| MQTL3.2 | LOC_Os03g41600 | DUF260 domain containing protein, putative | Yes | Yes | Yes | Yes | Yes |
| MQTL3.2 | LOC_Os03g42020 | calcium-transporting ATPase, plasma membrane-type, putative | Yes | Yes | Yes | Yes | Yes |
| MQTL3.2 | LOC_Os03g42100 | helix-loop-helix DNA-binding domain containing protein | Yes | Yes | Yes | Yes | Yes |
| MQTL3.2 | LOC_Os03g42110 | semialdehyde dehydrogenase, NAD binding domain containing protein, putative | Yes | Yes | Yes | Yes | Yes |
| MQTL3.2 | LOC_Os03g42200 | dof zinc finger domain containing protein, putative | Yes | Yes | Yes | Yes |  |
| MQTL3.2 | LOC_Os03g42290 | B3 DNA binding domain containing protein | Yes | Yes | Yes |  | Yes |
| MQTL3.3 | LOC_Os03g51330* | GRAS family transcription factor domain containing protein, | Yes | Yes | Yes | Yes | Yes |
