# Supplementary material for: Understanding complex genetic architecture of rice grain weight through QTL-meta analysis and candidate gene identification
Source: Sci Rep. 2022 Aug 16;12:13832. doi: 10.1038/s41598-022-17402-w (PMC9381546; doi:10.1038/s41598-022-17402-w)
Supplement: Supplementary file 9 — Supplementary Information 9. [file 41598_2022_17402_MOESM9_ESM.docx]

Supplementary file 6. Details of genotypes from 3K panel used for sequence difference analysis

| Sl. No. | 3K DNA unique ID | Accession name | origin | Grain weight (g) | Class |
| --- | --- | --- | --- | --- | --- |
| 1 | IRIS_313-10868 | ARC 11571 | India | 10.81 | Low grain weight |
| 2 | IRIS_313-11362 | KEYA NUNIA | India | 10.96 |  |
| 3 | IRIS_313-9629 | JC157 | India | 11.73 |  |
| 4 | IRIS_313-11826 | HIRA KHI | India | 12.61 |  |
| 5 | IRIS_313-11257 | ARC 12757 | India | 34.47 | High grain weight |
| 6 | IRIS_313-8147 | T 757 | India | 34.28 |  |
| 7 | IRIS_313-10884 | ARC 12493 | India | 33.08 |  |
| 8 | IRIS_313-11420 | PISINI | India | 31.94 |  |
